# Evaluation of the relative potential for contact and doffing transmission of SARS-CoV-2 by a range of personal protective equipment materials

**DOI:** 10.1038/s41598-022-20952-8

**Published:** 2022-10-05

**Authors:** Xuan Xue, Christopher M. Coleman, Joshua D. Duncan, Andrew L. Hook, Jonathan K. Ball, Cameron Alexander, Morgan R. Alexander

**Affiliations:** 1 Division of Advanced Materials and Healthcare Technologies, School of Pharmacy, University of Nottingham, Nottingham, NG7 2RD UK; 2 School of Life Sciences, University of Nottingham, Queen’s Medical Centre, Nottingham, NG7 2UH UK; 3 Wolfson Centre for Research on Global Virus Infections, University of Nottingham, Queen’s Medical Centre, Nottingham, NG7 2UH UK; 4 Nottingham Biomedical Research Centre, University of Nottingham, Queen’s Medical Centre, Nottingham, NG7 2UH UK; 5 Division of Molecular Therapeutics and Formulation, School of Pharmacy, University of Nottingham, Nottingham, NG7 2RD UK

**Keywords:** Microbiology, Health care, Chemistry, Materials science

## Abstract

Severe acute respiratory syndrome coronavirus 2 (SARS-CoV-2)—the causative agent of coronavirus disease 2019 (COVID-19)—has caused a global public health emergency. Personal protective equipment (PPE) is the primary defence against viral exposure in healthcare and community settings. However, the surfaces of PPE materials may trap virus for contact transmission or through laden aerosols generated during removal of PPE, through cleaning or during movement. In this study, the relative efficacy of current PPE materials in terms of virion adsorption to materials and their antiviral potency, has been evaluated on a wide range of PPE for the first time, including four polymer glove types, two types of scrubs, apron material, a mask, visor and a selection of other commercial polymers and products. Although differences in virion adsorption to the test materials were observed, none of the existing polymer-based PPE resulted in more than tenfold reduction in the SARS-CoV-2 titre within either 10 min or 30 min contact period. The wettability and surface chemistry of the test materials were analysed to investigate any correlations with their surface physicochemical properties. While no correlation was found between wettability and viral retention under air flow challenge, one secondary ion of m/z 101.03 (+) and three secondary ions of m/z 31.98 (−), 196.93 (−) and 394.33 (+) in ToF–SIMS data of the test materials showed positive and negative correlations with the viral retention, respectively, which was identified by PLS regression model, suggesting that the surface chemistry plays a role in determining the extent of virion adsorption. Our findings outline the material aspects that influence the efficacy of current PPE against SARS-CoV-2 transmission and give suggestions on the development of novel simple polymer-based PPE for better infection protection.

## Introduction

The world faces a severe and acute public health emergency due to the ongoing COVID-19 pandemic caused by SARS-CoV-2. Although the mortality rate has reduced due to the increasing immunity in the community from vaccination and infection, and possibly through virus attenuation, the infection control situation is still uncertain due to emerging variants. Like other human respiratory viruses, SARS-CoV-2 is envisaged to spread through direct inhalation of virus laden droplets or, more rarely, aerosols, or by hand contact with contaminated surfaces and subsequent transfer to mucus membranes^1^. Healthcare workers are in the front line of the COVID-19 outbreak response and are highly exposed to SARS-CoV-2. PPE is their main defence against viral contamination; disposable gloves, visors, face masks and gown materials are designed to act as a shield against viral transfer from infected patients^2^. However, virus-laden aerosols may be generated during removal (doffing) of PPE, through cleaning, or via the movement of staff^3–5^. Healthcare workers are, therefore, advised to be careful when changing their PPE, and consistently clean surfaces to prevent the spread of the virus.^6^

The persistence of SARS-CoV-2 on surfaces^7,8^ including some commonly used PPE^9^ has been discussed in earlier studies^10^. Under ambient conditions, live virus was found to remain detectable up to 4 h on copper, up to 24 h on cardboard, and up to 2–3 days on polypropylene and stainless steel^7^. Another study focused on PPE surfaces where SARS- CoV-2 remained viable for up to 7 days on nitrile gloves, 4 days on chemical-resistant gloves, 21 days on plastic face shield and N95/N100 particulate respirators, and 14 days on Tyvek® and stainless steel under ambient conditions^9^. SARS-CoV-2 infectivity on cotton was reduced within 4 h of drying and was not detectable after 24 h in the same study^9^. It is worth noting that without effective surface cleaning or changing PPE, SARS-CoV-2 virions persisted on such surfaces are likely to be relocated for further spreading even within the containment room in clinical settings^11^. The infectious dose of SARS-CoV-2, namely, the average number of viral particles required to establish an infection for COVID-19, is unknown, but can be as low as 10 tissue culture infective dose (TCID_50_) (1 log unit of TCID_50_) according to the recent SARS-CoV-2 human challenge study^12^. However, relatively high inoculation doses (> 5 log units of TCID_50_ on material piece) were used in these studies to achieve extended detectable values over the long test period.

To date, there is still limited information regarding the efficacy of current PPE materials in antiviral and virucidal activities. To be claimed as an antiviral material, the material should be able to inactivate the viruses within a short period, for example by a 4 log reduction of virus titre within 30 min^13^. Researchers often use TCID_50_ endpoint titration and viral plaque assay to quantify the live virus recovered from the material surface within the test period to determine the antiviral/virucidal efficacy of the polymeric coatings^13,14^ or PPE^15,16^. Relatively high inoculation doses were used in these studies (> 3 log units of plaque forming units (pfu) on material piece^13,14^). However, different research groups have used different starting points.

In this study, we aimed to evaluate the antiviral efficacy of a range of existing PPE (gloves, scrubs, visor and face masks) and common work surfaces via measuring live SARS-CoV-2 viral lifetimes for short surface residence timescales of relevance to viral contact with these surfaces and their resuspension by doffing in the clinic. The possibility of spread of the viruses from PPE and surfaces using pseudo-virus particles was also assessed using a laminar air flow system to mimic the air flow generated by movement and doffing within the workplace. We investigated the role of the products surface physicochemical properties on viral retention, and therefore compared the viral binding with measured water contact angle and surface chemistry of the PPE to identify putative material surface chemistries correlating with increase viral binding across the clinical PPE sample set. Our findings outline the material aspects that influence the efficacy of current PPE against SARS-CoV-2 transmission. Suggestions have been made on the development of novel antiviral polymer-based PPE for better infection protection.

## Materials and methods

Products covering a range of widely used PPE materials during the COVID-19 pandemic, common plastics used in public settings, and a *Virustatic* shield fabric were obtained for this study. The ten PPE products were examined on separate sides when relevant, denoted: latex, nitrile, neoprene and vinyl gloves; polyethylene (PE) apron, scrub 1 (65% polyester 35% cotton), scrub 2 (100% cotton); 3-layer medical face mask (model: FM301; a polypropylene outer hydrophobic nonwoven spunbond layer (O), a polypropylene middle filter meltblown layer (M) and an inner soft absorbent nonwoven spunbond layer (I)); polyethylene terephthalate (PET) visor (non-coated outer side (O) and anti-fog coated inner side (I)); Virustatic shield^17,18^ with anti-viral protein coating; and four common plastic surfaces: polystyrene (PS), polyurethane (PU), polycarbonate (PC) and polytetrafluoroethylene (PTFE). The supplier and primary polymer component(s) for each of the test materials are summarised in Table 1.

**Table 1:** Summary of the PPE and other common materials being tested in this study, their suppliers and primary polymer components.

PPE/surface	Source	Polymer material	
		Primary surface component	
Latex gloves	FisherBrand™		
Nitrile gloves	Supreno		
Neoprene gloves	NeoTouch™		
Vinyl gloves	Ph Bodyguards		
PE apron	BPI		
Scrub 1 (65% polyester 35% cotton)	Fisher Scientific		
Scrub 2 ( 100% cotton)	UniMediForm		
Medical face mask	Intco	Outer layer	
		Middle layer	
		Inner layer	
PET visor	University of Nottingham, Engineering	Outer side	
		Inner side	
Virustatic shield	Virustatic Ltd	Anti-viral cationic protein coated fabric	
PS	Thermo Scientific		
PU	GoodFellow		
PC	Merck Life Science		
PTFE	Merck Life Science		

### Cells

Vero E6 cells were a kind gift from Prof. Kin-Chow Chang, Department of Veterinary Medicine and Science, University of Nottingham were maintained in minimal essential media supplemented with heat inactivated foetal calf serum, 2 mM l-glutamine and 1% penicillin/streptomycin (all from Sigma). Cells were maintained at 37 °C in 5% CO_2_ in an incubator before and during experiments.

### SARS-CoV-2 live virus

SARS-CoV-2 (CVR-GLA-1 variant) was obtained from the Centre for AIDS Reagents, NIBSC. Viral stocks were created by propagation of the original virus in Vero E6 cells and quantified using the median TCID_50_ method as previously described for other coronaviruses^19^. All work with live SARS-CoV-2 was performed under containment level 3 conditions at the University of Nottingham.

### Determination of antiviral efficacy of material surfaces against SARS-CoV-2

A small piece of each material (1 × 1 cm^2^) was excised from the products and placed in the well of a 96-well tissue culture treated polystyrene plate (Corning). 10 mL of virus stock containing 7.2 × 10^3^ TCID_50_ of SARS-CoV-2 was added to each material piece and incubated for 10 or 30 min at room temperature and ambient humidity within a Class I/III microbiological safety cabinet with the normal airflow engaged, in a containment level 3 laboratory under negative pressure. These study conditions have been chosen to mimic as closely as possible the real-life time periods for exposure of PPE to viral particles suspended in liquid. The lid of the 96-well plate was in place for the duration of the incubation. After 10 or 30 min, the surface was washed with 200 mL fresh Vero E6 cell growth media and the levels of SARS-CoV-2 recovered were quantified using the TCID_50_ method.

### Real-time RT-PCR assay

After the incubation period, the media was added to TRIzol reagent (Ambion) at a 1:1 ratio and the remaining material was submerged in 500 mL of TRIzol reagent (Ambion). RNA was extracted using the DirectZol kit (Zymo Research) according to the manufacturers’ instructions. SARS-CoV-2 RNA was quantified using primers targeted to the RNA-dependent-RNA polymerase^20^. SARS-CoV-2 RNA was assessed using the QuantiNova® SYBR ® Green RT-PCR kit (Qiagen) and a FAST 7500 Real-Time PCR System (Applied Biosystems), both according to the manufacturers’ instructions. Relative expression was determined using the deltaCt method, compared to PS control.

### Air flow detachment of SARS-CoV-2 pseudo-virus particles from material surfaces

Fluorescently labelled retroviral SARS-CoV-2 pseudo-virus particles were produced as an alternative to live virus, to allow for the testing of surfaces outside of a containment level 3 environment. SARS-CoV-2 retroviral pseudo-virus particles were expressed in HEK 293t cells as previously described^21^. Specifically, 1.5 × 10^6^ cells were seeded in a 10 cm Primaria coated plate (Corning) in 5 mL of Dulbecco’s modified eagle medium (DMEM) (Gibco) supplemented with 10% heat inactivated foetal bovine serum (Sigma) and 1% non-essential amino acids (Gibco) and incubated overnight at 37 ºC in 5% CO_2_. Cells were transfected with 2 µg murine leukemic virus gag-pol (phCMV-5349) packaging vector and 2 µg pcDNA3.1 plasmid encoding SARS-CoV2 Spike (Wuhan isolate). Plasmids were incubated with 24 µg polyethylenime (Polysicences) in a total volume of 600 µL of OptiMEM (Gibco) for 1 h at room temperature. The DMEM was replaced with 7 mL of OptiMEM and plasmids were added to the dish and incubated at 37 ºC, 5% CO_2_ for 6 h. The OptiMEM was replaced with 10 mL DMEM and cells grown for 72 h. Cell supernatant was harvested at 48 h and fresh DMEM was added followed by a final harvest at 72 h post transfection. Supernatant was clarified by centrifugation at 3000×*g* for 20 min and passed through a 0.45 µM syringe filter. Pseudo-virus particles were concentrated by ultracentrifugation using the sucrose cushion method as described previously^22^. Briefly, 20 mL of clarified cell supernatant was overlayed onto a 3 mL 20% sucrose cushion in a 26.3 mL polycarbonate ultracentrifuge tube (Beckman Coulter). Samples were placed in a fixed angle Type 70 Ti rotor (Beckman Coulter) and centrifuged at 110,000×*g* for 2.5 h. Supernatant was removed and pelleted pseudo-virus particles were resuspended overnight at 4 °C in 200 µL sterile Dulbecco’s phosphate buffered saline (PBS). Purified particles were quantified by total protein concentration using the microBCA assay kit (Thermo-Scientific). Pseudo-virus particles were fluorescently tagged using the Alexa Flour 555 protein labelling kit (Thermo-Scientific) and stored at − 20 °C.

Each sample coupon (1 cm^2^) was fixed on one microslide. 2 mL of 0.5 mg/mL Alexa Fluor 555 tagged pseudo-virus particles in PBS solution was deposited on the centre of each coupon and allowed to dry under ambient condition (20–22 °C,  ~ 50% RH) for 30 min. An air flow generator (3b Scientific Ltd, UK) with air nozzle (3b Scientific Ltd, UK) to generate laminar air flow (5.6 m/s) was used to blowing the pseudo-virus particles on the material surfaces oriented approximately parallel to the surface. Fluorescence images of the area both before and after applying the air flow for 10 min, 30 min and 1 h were acquired using an automated fluorescence microscope IMSTAR (PathFinder™, France) and processed using Fiji Image J software (version 2017 for macOS). A composite image (autofluorescence was subtracted from the material area without the pseudo-virus particles solution) was cropped using a circle to the border of the spots to determine the fluorescence intensity per pixel due to pseudo-virus binding.

### Water contact angle measurement of material surfaces

Static contact angles using deionized water were measured on material samples (Table 1) using a CAM 200 Optical Contact Angle Meter (KSV Instruments Ltd, UK) under ambient condition (20–22 °C,  ~ 50% RH). At least three measurements were recorded for each sample and the mean and standard deviation were determined in each case.

### Time-of-flight secondary ion mass spectrometry (ToF–SIMS) analysis of sample surfaces

Material samples were cut into 1 cm^2^ coupons and measured by ToF–SIMS as received from manufacturer without additional processing. ToF–SIMS data were collected using a ToF–SIMS IV instrument (ION-TOF GmbH., Münster, Germany) equipped with a bismuth liquid metal ion gun and a single-stage reflectron analyser. Bi_3_^+^ primary ion energy of 25 kV and a pulsed target current of approximately 1.3 pA were used in this measurement. Low-energy electrons (20 eV) were used to compensate for surface charging due to the positively charged primary ion beam on the insulating surfaces. Rastered areas of 3 × 3 mm^2^ were analysed at a resolution of 100 pixels per mm and 15 frames per patch. The total primary ion beam dose for each analysed area was kept below 1 × 10^12^ ions per cm^2^, ensuring *static SIMS* acquisition conditions. Data acquisition and analysis were performed using IONTOF SurfaceLab7 software (IONTOF, Münster, Germany).

### Statistical analysis

Graphical representations of all experimental results, including averages, standard deviations, one-way ANOVA Dunnett’s and Tukey’s tests, and Student t-test, were performed using GraphPad Prism (version 9) and Microsoft Excel software (version 16.59). Biological replicates with no recoverable virus were assigned a value of zero for the purposes of these calculations. Partial least square (PLS) regression was performed as previously described^23^.

## Results

### Antiviral efficacy of material surfaces against SARS-CoV-2

**Figure 1:**
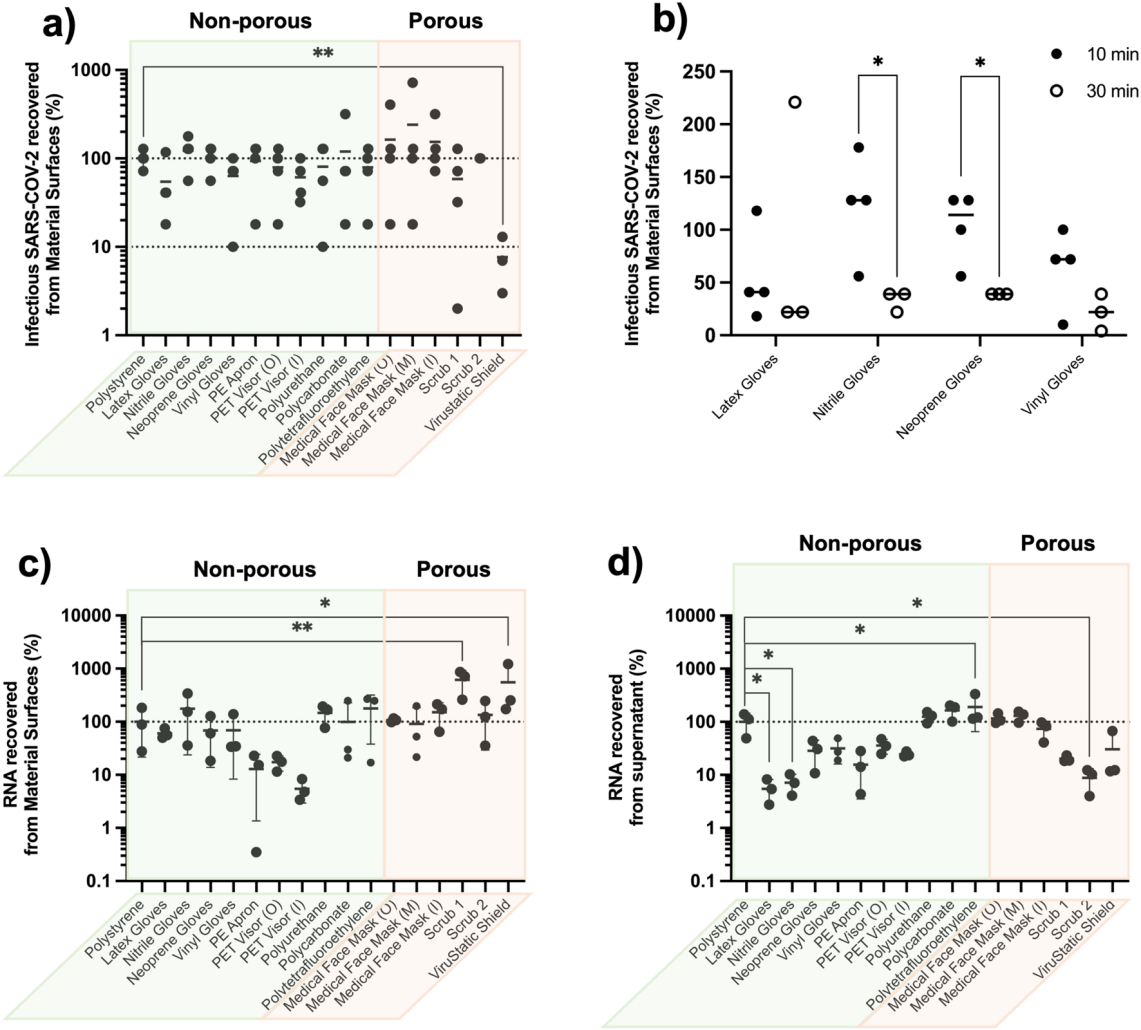
Antiviral efficacy of material surfaces evaluated by experimental inoculation of SARS-CoV-2 for (**a**) 10 min and (**b**) 30 min incubation (n ≥ 3). The viral load recovered from the surfaces was quantified by TCID_50_ endpoint titration and viral plaque assay; one dot represents the result obtained from one experimental repeat. Significant difference was found between PS and Virustatic shield (**P < 0.01) in 10 min incubation by one-way ANOVA Dunnett’s test, between 10 and 30 min incubation of nitril gloves (*P < 0.05), and between 10 and 30 min incubation of neoprene gloves (*P < 0.05) by Student *t* test. The relative amount of SARS-CoV-2 RNA recovered by elution from (**c**) the surfaces and (**d**) the supernatant was quantified by Real-Time PCR (n = 3); one dot represents the result obtained from one experimental repeat. Significant difference was found between PS and the materials of scrub 1 (**P < 0.01) and Virustatic shield (*P < 0.05) on material surfaces by one-way ANOVA Dunnett’s test, and between PS and the materials of latex gloves (*P < 0.05), nitrile gloves (*P < 0.05), PTFE (*P < 0.05) and scrub 2 (*P < 0.05) in supernatant by one-way ANOVA Dunnett’s test.

Virus stock containing 7.2 × 10^3^ (equals to 3.9 log units) TCID_50_ of SARS-CoV-2 was inoculated on each 1 × 1 cm^2^ material piece. After 10 min incubation under ambient condition, the viral load recovered from each material was determined by TCID_50_ assay, and compared to a PS surface (non-treated tissue culture well plate), as the control to give a percentage of viral degradation attributed to the presence of the material sample (Fig. 1a,b). By doing this, any loss of virus due to experimental procedures and natural virus decay outside a host was taken into account.

After 10 min incubation, the proportions of viable SARS-CoV-2 recovered from latex, nitrile, neoprene and vinyl gloves were 54.5%, 122.5%, 103.0% and 63.5%, respectively, while those of PE apron, scrub 1 (65% polyester, 35% cotton), and scrub 2 (100% cotton) were 93.5%, 58.5% and 100.0%, respectively. The results of PET inner and outer sides were 79.5% and 61.3%, respectively. For the 3-layer medical face masks, these numbers were 162.8%, 241.5% and 154.3%, respectively, for outer, middle and inner layers (Fig. 1a) . In brief, the proportions of viable SARS-CoV-2 recovered from all test materials were calculated and then analysed using one-way ANOVO Dunnett’s multiple comparisons test to compare with the PS control, and to evaluate the statistical difference. No significant loss of SARS-CoV-2 was observed following contact with any of these material surfaces, except of “Virustatic shield” (a commercially available protein coated woven fabric) where recovered viable SARS-CoV-2 reduced to an average of 7.7% (*P < 0.01) within 10 min test period.

It has been widely reported that the persistence of SARS-CoV-2 on surfaces of PPE and common surface materials decayed over time^8–10^. Contact time is, therefore, a key component of antiviral activity, and 10 min may be too short for a significant effect to be observed. We then performed the same assay with the four types of gloves for a prolonged period of 30 min incubation before recovering and quantifying the viable viruses from the surfaces. The results were compared to 10 min incubation data of the same materials. As shown in Fig. 1b, the levels of viable SARS-CoV-2 recovered from latex gloves and vinyl gloves after 30 min incubation period were not significantly different from those obtained after 10 min incubation. Moreover, a significant difference was observed for nitrile gloves and neoprene gloves, where the number of infectious viruses recovered decreased from 122.5 to 33.3% for nitril gloves (*P < 0.05 by student *t* test), and from 103.0 to 39.0% for neoprene gloves (*P < 0.05 by student t-test).

To better understand the surface adsorbed status of virions and that of viral RNA through the experiment, the same experimental settings as for the TCID_50_ assay over a 10 min incubation period were performed with the test materials, and the relative levels of viral RNA extracted from both material surfaces and supernatants were quantified using a SARS-CoV-2 specific RT-PCR assay (Fig. 1c,d) . The relative level of viral RNA extracted from Virustatic shield surfaces was 551%, namely 5^1/2^ times of that of PS, on average of the three biological repeats (Fig. 1c). The lower titre (**P < 0.01) (Fig. 1a) and higher RNA level (*P < 0.05), (Fig. 1c) compared to the PS control by one-way ANOVA Dunnett’s test, observed on the Virustatic shield indicated that the virions were being bound to the surface, and free viral RNA were possibly present on the material surfaces. Again, the levels of relative RNA extracted from scrub 1 were found to be high, indicating the presence of free viral RNA outside virions. All other test materials were not significantly different from the PS control, (Fig. 1c) although reduced viral RNA levels were observed on PE apron (12.9%), PET visor outer side (17.3%) and inner side (5.5%), which indicated that the viral RNA is possibly, to some extent, being degraded on these surfaces. Additionally, although the relative levels of RNA extracted from latex gloves, nitrile gloves, neoprene gloves and vinyl gloves surfaces were not significantly different from the PS control, which were 60.7%, 176.5%, 68.8% and 69.1%, respectively; those of the supernatants were 5.5%, 7.1%, 28.4% and 31.7%, respectively, where viral RNA levels were significantly reduced in the supernatants of latex gloves (*P < 0.05) and nitrile gloves (*P < 0.05) (Fig. 1d). Similarly, reduced levels of viral RNA were observed from the supernatants of Scrub 2 (*P < 0.05). Again, this possibly indicated that the viral RNA was being degraded in the supernatant. Also, the high level of viral RNA in PTFE supernatant may indicate the presence of free viral RNA outside a virion. Moreover, no significant changes in either TCID_50_ assay or relative RNA levels of both surface and supernatant were shown in neoprene gloves, vinyl gloves, PE apron, PET visors (both sides), PU, PC, and medical face masks (all three layers), which suggested that these materials possibly do not give any effect on SARS-CoV-2 in contact with them.

### Detachment of SARS-CoV-2 pseudo-virus particles from material surfaces

**Figure 2:**
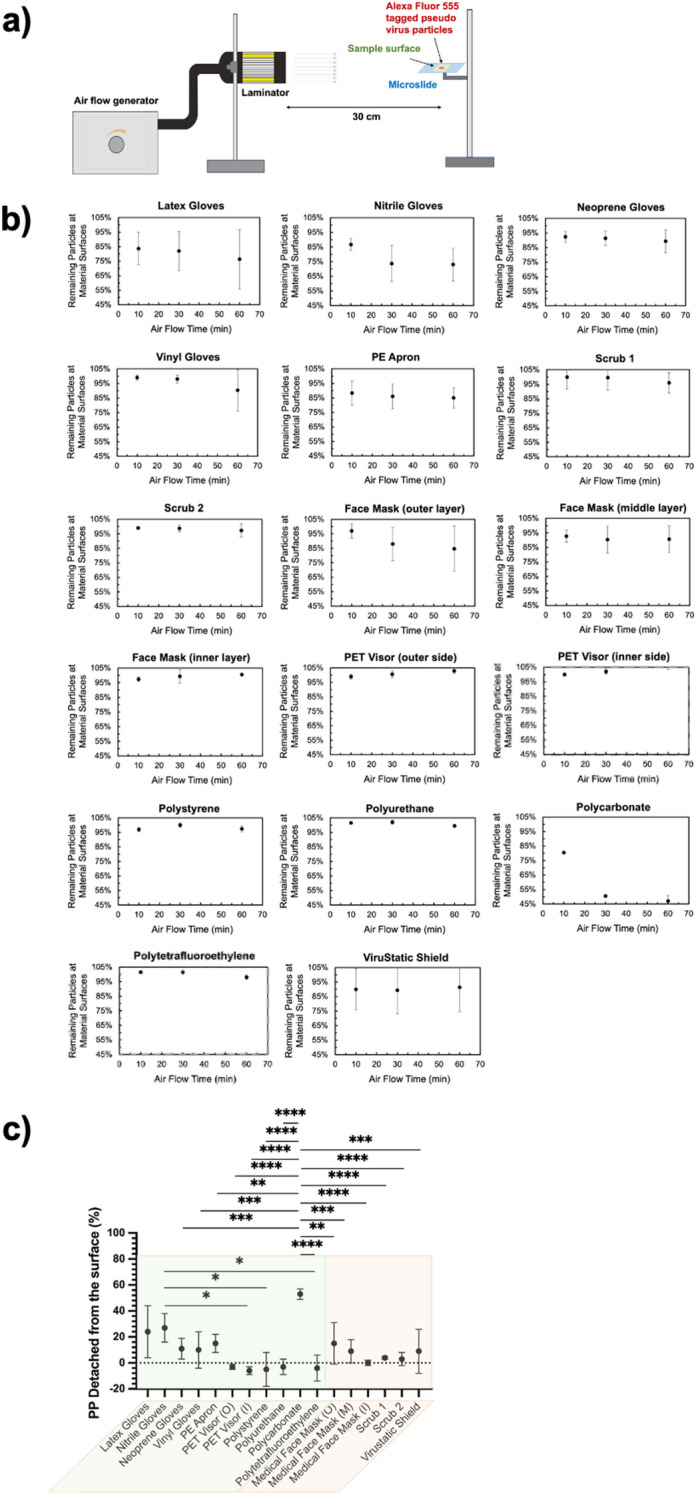
(**a**) The schematic description of the air flow detachment experimental setup. (**b**) The average fluorescence intensity represents the number of pseudo-virus binding on the surfaces. The numbers of pseudo-virus particle remaining on the surface after applying air flow for 10 min, 30 min and 1 h were compared to that before applying the air flow at time zero (100%). (n = 5) (c) The comparison of the percentage of detached pseudo-virus particles, represented by the average fluorescence intensity, from the sample surfaces after applying air flow for 1 h. *P* values from one-way ANOVA Tukey’s test are shown. *P < 0.05, **P < 0.01, ***P < 0.001, ****P < 0.0001.

We designed an experiment applying 5.6 m/s air flow from the side of a horizontal face-up surface with a laminar air flow intended to mimic the scenario of doffing and movement in a working environment (Fig. 2a). The air flow rate applied in a fume cupboard in the lab was ~ 0.48 m/s, which is higher than that of other vented areas in the lab and in the office. Thus, particles not removed under such strong air flow challenge it is unlikely they would be removed in a real workplace environment. Non-infectious SARS-CoV-2 pseudo-virus particles with similar structural features and shape to live SARS-CoV-2 virions were used in this experiment to allow it to be performed outside containment level 3 and with constitutive fluorescence. A 2 mL drop of 0.5 mg/mL Alexa Fluor 555 tagged pseudo-virus particles in PBS solution was deposited on materials, where the large inoculation volume was selected to mimic the frequent release and large spread of virus laden droplet on PPE and surfaces in clinical settings. The droplet was allowed to dry in the dark under ambient condition for 30 min before the first microscope image was acquired. Fluorescence images of the centre area before and after applying an air flow for 10 min, 30 min and 1 h were acquired to record the fluorescence from the pseudo-virus particles retained on the surface as an approximation of the retention of particles on the different surfaces. The average fluorescence intensity represents the relative amount of pseudo-virus particles on the surfaces. The relative amount of pseudo-virus particles retaining on the surface after applying air flow for 10 min, 30 min and 1 h were compared to that before air flow challenge.

SARS-CoV-2 pseudo-virus particles exhibited strong binding to scrubs, medical face mask inner layer, PET visor (both sides), PS, PU and PTFE. The florescence from pseudo-virus particles retaining on the surface was above 95% of the initial value under laminar air flow for up to 1 h (Fig. 2b). In contrast, the medical face mask outer and middle layers showed reduced binding affinity compared to the inner layer, but the relative pseudo-virus particle amount were still above 85% and 91% after 1 h, respectively. The four types of gloves exhibited different retention of SARS-CoV-2 pseudo-virus particles. The relative pseudo-virus particle amount on latex gloves dropped to 84% within 10 min of air flow challenge, and gradually reduced to 82% in 30 min and 76% in 1 h. Similar retention was observed on nitrile gloves; from 87% in 10 min, 74% in 30 min to 73% in 1 h. However, Neoprene and vinyl gloves both retained 90% pseudo-virus particles after 1 h, which indicated strong viral retention under airflow challenge. For PE apron, the relative amount of pseudo-virus particle dropped to 88% in 10 min, but remained around 85% after 1 h. PC showed the least binding to SARS-CoV-2 pseudo-virus particles among all the materials. The relative amount of pseudo-virus particles retained dropped dramatically to 81% after 10 min, followed by another significant drop to 51% after 30 min, and stayed around 47% after 1 h. The antiviral Virustatic shield exhibited similar strength for pseudo-virus particles adhesion compared to neoprene gloves and medical face mask middle layer, which retained about 90% pseudo-virus particles through the 1 h test period.

To better understand the pseudo-virus binding against different materials, the proportion of pseudo-virus detached and removed under 1 h air flow challenge were calculated and compared in Fig. 2c. One-way ANOVA Tukey’s test was performed to analyse the statistical differences between any two types of materials, where significant differences between PC and all other materials, except of latex gloves and nitrile gloves, were observed and shown in Fig. 2c (*P < 0.05, **P < 0.01, ***P < 0.001, ****P < 0.0001). In addition, we found significant difference between nitrile gloves and three other materials including PET visor inner side (*P < 0.05), PS (*P < 0.05) and PTFE (*P < 0.05). These experimental results indicate the material aspects that influence the retention of SARS-CoV-2 particles on current PPE and other common material surfaces under air flow challenge.

### Surface wettability

**Figure 3:**
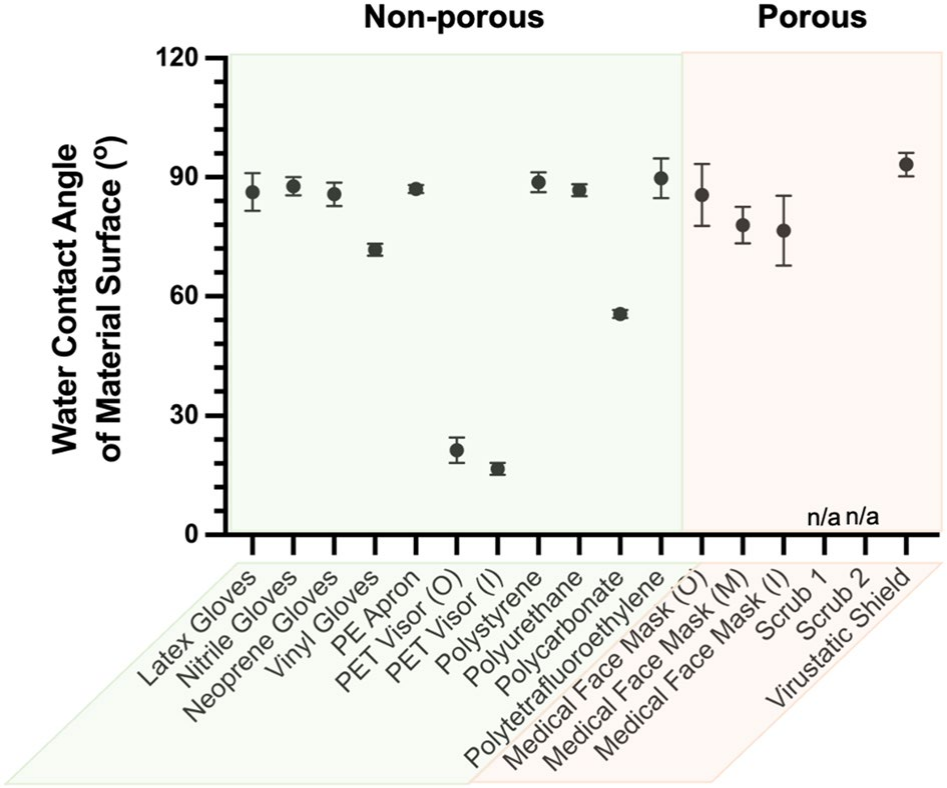
Water contact Angles for material surfaces measured under ambient condition (20–22 °C,  ~ 50% RH) (n = 3).

The water contact angles of all test materials were measured as an estimate of their physicochemical surface properties, plotted in Fig. 3 and Table S1 in ESI. The correlation between the material surface wettability and the pseudo-virus particle retention under air flow challenge were plotted, (Fig. S1) where no direct correlation was found. For instance, latex, nitrile and neoprene gloves all had similar surface wettability (Fig. 3) but only neoprene gloves showed a high pseudo-virus particle retention (Fig. 2a,b). Similarly, neoprene and vinyl gloves both had similar pseudo-virus particle retention (Fig. 2a,b) but different surface wettability (Fig. 3).

### Chemical compositions of the surfaces by ToF–SIMS

To interpret the viral interactions with the products, the surface chemistry was probed using ToF SIMS.

**Figure 4:**
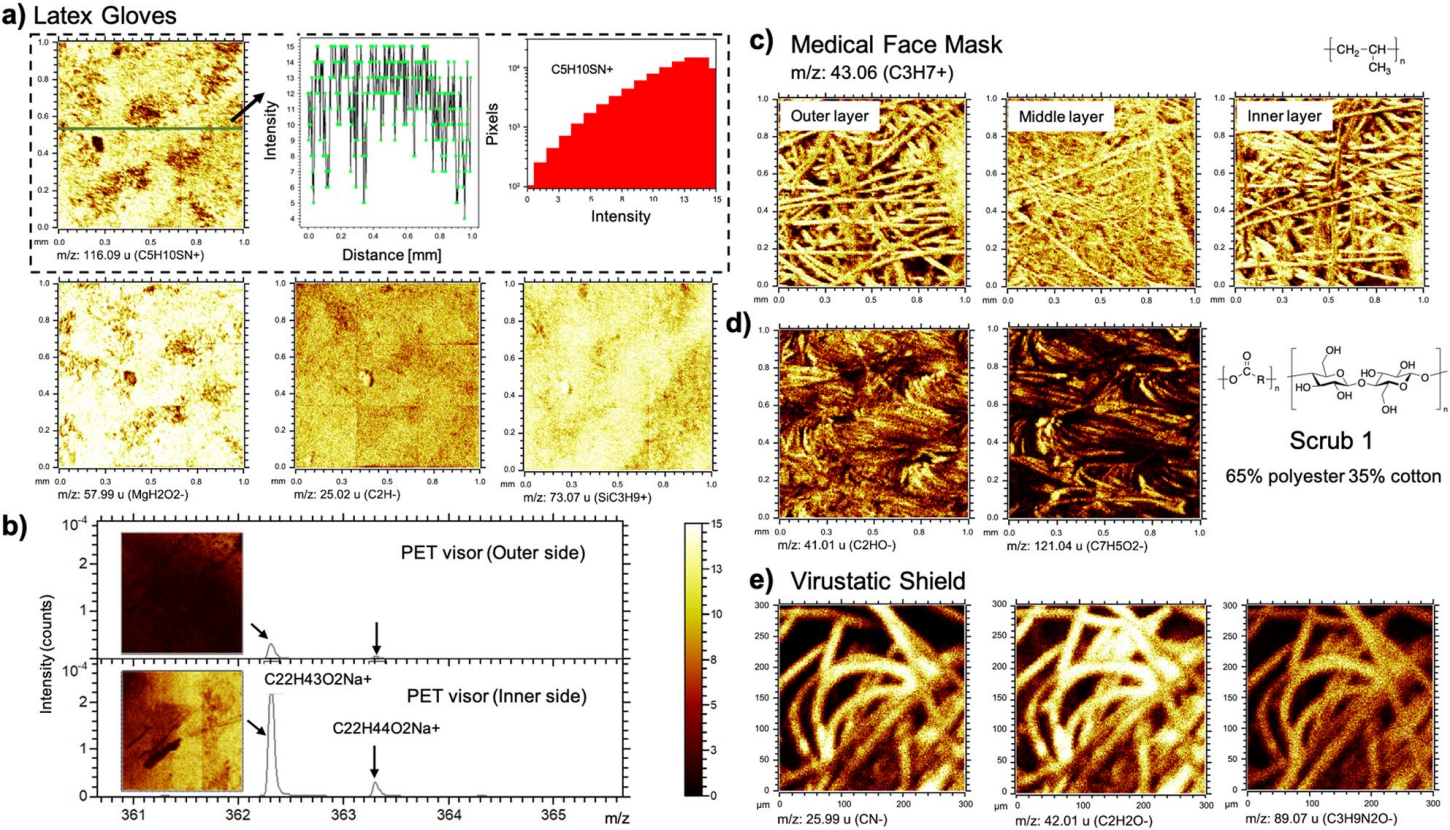
Corresponding ion images acquired using ToF–SIMS from surfaces of (**a**) latex gloves, (**b**) PET visor, (**c**) medical face mask, (**d**) scrub1 and (**e**) Virustatic shield. In (**a**), the profile of C_5_H_10_SN^+^ (m/z 116.09) intensity as a function of the distance and the histogram of the pixels for intensity indicate the non-uniform distribution of corresponding component on the surface. In (**b**), the ToF–SIMS spectra of PET visor outer and inner sides indicate the ion peaks at m/z 362.3 and 363.3 only identified on the outermost surface of the inner side were putatively assigned to C_22_H_43_O_2_Na^+^ and C_22_H_44_O_2_Na^+^ and possibly from the component of the anti-fog agent.

Latex gloves, made of natural rubbers with polyisoprene as their primary chemical constituent, are one of the most commonly used types of medical gloves in healthcare settings^24^. Non-specific hydrocarbon secondary ions from the aliphatic backbone of polyisoprene were identified as C_2_H_3_^+^, C_2_H_5_^+^, C_3_H_3_^+^, C_3_H_5_^+^, C_3_H_7_^+^, C_4_H_7_^+^, C_4_H_9_^+^ in the positive spectrum and CH^−^, C_2_H^−^ in the negative spectrum. The representative secondary ions of C_3_H_7_O^+^ (m/z 59.04) and C_4_H_10_O^+^ (m/z 74.07) were intensive signals putatively attributed to propylene glycol surfactant^25^, which combines with the rubber emulsion to enhance the colloidal properties of the rubber latex^26^. There were also intensive signals found at m/z 59.99, 88.02 and 116.09, which are putatively assigned to CH_2_SN^+^ (or CH_2_NO_2_^+^), C_3_H_6_SN^+^ (or C_3_H_6_NO_2_^+^), and C_5_H_10_SN^+^ (or C_5_H_10_NO_2_^+^) from the commonly used rubber accelerators (e.g. thiourea or carbamate)^27^. Due to the mixture process in manufacturing, the distribution of the ions on the surface was not uniform as observed in the ToF–SIMS images. (Fig. 4a) The ions of SiC_3_H_9_^+^ (m/z 73.07) and Si_2_C_5_H_15_O^+^ (m/z 147.11) were also intensive signals commonly identified on PPE samples and are representative signals for poly(dimethyl siloxane) (PDMS). (Fig. 4a) Salt ions, including Na^+^, Ca^+^, K^+^, were also found widely in our PPE samples. Additionally, MgH_2_O_2_^−^ (m/z 57.99) ion was found in the negative spectrum, which we believe is due to the addition of talc silicate (Mg_3_Si_4_O_10_(OH)_2_) in the manufacturing process (Fig. 4a).

Nitrile gloves are mainly made of a synthetic copolymer of acrylonitrile and butadiene monomers. The secondary ions for the primary constituents were CH_3_^+^, C_2_H_3_^+^, C_2_H_4_^+^, C_2_H_5_^+^, C_3_H_5_^+^, C_3_H_7_^+^, C_4_H_5_^+^, C_4_H_7_^+^, C_4_H_9_^+^, C_5_H_5_^+^, C_5_H_7_^+^, C_5_H_9_^+^ and CNK^+^ in the positive spectrum, and CN^−^, C_2_H^−^ and CN^−^ in the negative spectrum. The same caveat for the specificity of these ions applied as for the latex gloves, although the nitrogen containing fragments do support the presence of acrylonitrile. Sulfur containing additives were possibly involved in the manufacturing process of these gloves as evidenced by CH_5_S^−^, SO_3_^−^, and SO_3_H^−^ ion signals in the negative spectrum. A series of phenyl group related ions, for example C_6_H_5_^+^, C_6_H_7_^+^, C_6_H_9_^+^ and C_7_H_7_^+^, were found in the positive spectrum. Salt ions of Na^+^, Ca^+^ and K^+^ were identified. Particularly, Ca^+^ signal was observed to be intensive and, therefore, the secondary ion at m/z 112.92 was putatively assigned to a calcium containing component Ca_2_O_2_H^+^ or Ca_2_HS^+^.

The primary constituent of neoprene gloves is polychloroprene, which was identified with the backbone ion signals of C_2_H_3_^+^, C_2_H_5_^+^, C_3_H_5_^+^, C_4_H_7_^+^, C_4_H_9_^+^, C_5_H_7_^+^, C_5_H_9_^+^ in the positive spectrum and CH^−^, C_2_H^−^, Cl^−^, C_3_H_2_^−^, and C_2_H_3_Cl^−^ in the negative spectrum. PDMS was found in these glove surface as evidenced by the ions of SiC_3_H_9_^+^ and Si_2_C_5_H_15_O^+^. Salt ions of Na^+^, Ca^+^ and K^+^ were identified.

The primary component of vinyl gloves is poly(vinyl chloride) and was identified with the ions of C_2_H_3_^+^, C_2_H_5_^+^, C_3_H_5_^+^, C_3_H_7_^+^, C_4_H_5_^+^, C_4_H_7_^+^, C_4_H_9_^+^, C_5_H_11_^+^ in the positive spectrum and CH^−^, C_2_H^−^, and Cl^−^ in the negative spectrum. The ion signal found at m/z 149.02 was putatively assigned to C_8_H_7_NO_2_ + which may be from the carbamate (–CO_2_N) group of the rubber accelerator. The sulfur ions, SO_3_^−^ and SO_4_H^−^, were identified in the negative spectrum attributed to the additives added. Again, the ions of SiC_3_H_9_^+^ and Si_2_C_5_H_15_O^+^ were found in the positive spectrum and possibly from PDMS introduced by the manufacturing process. Salt ions of Na^+^, Ca^+^ and K^+^ were identified. A K^+^ dust particle happened to be captured in the ToF–SIMS image, indicating that unexpected contaminants may attach to the PPE surface during the entire manufacturing, packaging, and distribution process before delivered to the end user.

The PET visor is primarily made of polyethylene terephthalate with anti-fog agent coated on the inner side. Herein, the primary surface component of the outer side and inner side were observed to be similar, with C_2_H_3_^+^, C_2_H_5_^+^, CH_3_O^+^, C_3_H_5_^+^, C_3_H_7_^+^, C_2_H_5_O^+^ (m/z 45.03), C_4_H_7_^+^, C_4_H_9_^+^, C_7_H_4_O^+^ (m/z 104.02) and C_7_H_5_O^+^ (m/z 105.03) found in the positive spectrum and C_2_H_3_O^−^ (m/z 43.02), CHO_2_^−^ (m/z 44.99), C_4_H^−^ (m/z 49.01), C_6_H^−^ (m/z 73.01), C_6_H_4_^−^ (m/z 76.03), C_7_H_4_O_2_^−^ (m/z 120.02) and C_7_H_5_O_2_^−^ (m/z 121.04) found in the negative spectrum. Propylene glycol surfactant were possibly used in the manufacturing process as evidenced by the secondary ions of C_3_H_7_O^+^ (m/z 59.05), C_4_H_9_O^+^ (m/z 73.05) and C_5_H_12_O^+^ (m/z 88.08) shown in the positive spectrum. However, the secondary ion signal at m/z 88.08 was more intensive on the visor’s inner side than that on the outer side, which is possibly attributed to the anti-fog agent coated on the inner surface and putatively assigned to C_4_H_8_O_2_^+^. Similarly, the signal found at m/z 362.32 and 363.32 on the outermost surface of the inner side of the visor is, therefore, due to the coating of the same compound and putatively assigned to C_22_H_43_O_2_Na^+^ and C_22_H_44_O_2_Na^+^ (Fig. 4b) Again, PDMS (SiC_3_H_9_^+^ and Si_2_C_5_H_15_O^+^) and salt ions (Na^+^ and K^+^) were identified on these surfaces.

The three layers of the medical face mask are all made of polypropylene. The ToF–SIMS spectra of all the three layers have been measured and showed similar signals with the characteristic secondary ions of polypropylene C_2_H_3_^+^, C_2_H_5_^+^, C_3_H_3_^+^, C_3_H_5_^+^, C_3_H_7_^+^, C_4_H_5_^+^, C_4_H_7_^+^, C_4_H_9_^+^, C_5_H_9_^+^, C_5_H_11_^+^, C_6_H_5_^+^ identified in the positive spectra, CH^−^ and C_2_H^−^ in negative spectra (Fig. 4c). The ion signals from other components (e.g. additives, salts, other contaminants) were not intensive for these PPE samples.

The PE apron in this study was primary made of polyethylene possibly with Nylon (or similar chemical structures) mixed in the manufacturing process as evidenced by the secondary ions of C_2_H_3_^+^, C_2_H_5_^+^, C_3_H_5_^+^, CH_2_NO^+^ (m/z 44.01), C_4_H_7_^+^, C_4_H_9_^+^, C_2_H_5_NO^+^ (m/z 59.04), C_5_H_7_^+^, C_5_H_9_^+^, C_3_H_6_NO^+^ (m/z 72.04) identified in the positive spectrum, and O^−^ (m/z 16.00), OH^−^ (m/z 17.00), CH^−^, C_2_H^−^, CN^−^ (m/z 26.01), C_2_H_2_O^−^ (m/z 42.01), CNF^−^ (m/z 45.01) and C_9_H_17_O_2_^−^ (m/z 157.13) found in the negative spectrum. The ToF–SIMS spectra of scrub 1 (65% polyester 35% cotton) and scrub 2 (100% cotton) were observed to give similar signals, while C_2_HO^−^, C_7_H_4_O_2_^−^ and C_7_H_5_O_2_^−^ ions were only identified in scrub 1 possibly due to the mixture of polyester in the raw material, (Fig. 4d) Mg^+^, MgNH_3_^−^ were only seen in scrub 2 and possibly introduced in the manufacturing process. PDMS and salt ion signals were identified on all these three PPE samples.

The commercial Virustatic shield surfaces are known to have been coated with a cationic protein similar to those found on the human upper respiratory tract to protect individuals from infection. Protein related secondary ions, including CN^−^ (m/z 26.01), C_2_H_2_O^−^ (m/z 42.01), and C_3_H_9_N_2_O^−^ (m/z 89.07), were found in the ToF–SIMS spectrum, (Fig. 4e) which confirmed the existence of the antiviral coating on this PPE surface. PDMS and salt ion signals were identified.

The full ToF–SIMS spectra and the putative assignments for PPE and other surface samples covered in this study are detailed in Figure S2 in ESI.

## Discussion

The persistence of SARS-CoV-2 on inoculated surfaces of PPE and common surface materials has been studied under various environmental conditions over prolonged periods of time^7–9^. However, there is limited information regarding the efficacy of current PPE in antiviral and virucidal activities, which usually require a drastic log reduction of viable viruses within a short contact period, for example 4 logs reduction of virus titre within 30 min in contact with the material^13^. The ISO 21702:2019 is the international standard for measurement of antiviral activity on plastics and other non-porous surfaces^28^, and is applied for certification of “antiviral” claims^17,29^. In addition, the standard within EN14885:2018 is a guide for testing the antiviral efficacy of materials to be used in a strict clinical setting, which supports virucidal efficacy claim of disinfectants used in the medical area with requirement to give at least 4 log reductions of viruses within 5 min for surfaces near patients or staff or 60 min for other surfaces^30^. In this study, the inoculation dose and incubation time were chosen based on the previous studies on antiviral materials^13,14^. For our own purposes, an antiviral material was determined to be a material that resulted in more than a tenfold reduction in the SARS-CoV-2 titre, as this is one full row of TCID_50_ assay. In other words, after incubation, the number of viruses recovered from PS is set to be 100%, and the virus recovered from other material surface were compared to that. If that is less than 10%, it means that material is antiviral. The Virustatic shield was the only material sample that gave a higher than tenfold reduction and thus claimed as antiviral SARS-CoV-2 PPE under this experimental setting. In particular, none of the existing simple polymer-based PPE can be claimed as antiviral against SARS-CoV-2, which if antiviral in nature would have advantages over the active-loaded and protein-coated products currently available as they deplete or degrade over time.

SARS-CoV-2 can contaminate surfaces via virus laden droplets, which may spread again as aerosols by the doffing (removing) of PPE, through cleaning, or via the movement of staff, so there could be a benefit to a surface which encourages strong binding^3–5^. The surface interaction and adhesion energy of spike protein, the structural and cell receptor binding protein on the outer surface of SARS-CoV-2, with polystyrene, stainless steel, gold and glass was quantified via atomic force microscopy by Xie et al.^31^ However, there is still limited information indicating the adsorption of SARS-CoV-2 particles on various PPE and material surfaces. In this study, the antiviral efficacy of the current PPE and common work surfaces with live SARS-CoV-2 and the possibility of spread of SARS-CoV-2 pseudo-virus particles from the contaminated material surfaces have been evaluated under ambient condition, which indicates the limitations of the current PPE in preventing SARS-CoV-2 fomite transmission.

To give suggestions on improving PPE and achieving high protection against SARS-CoV-2 for healthcare settings, the surface physicochemical properties of the test materials should be understood in detail, which relates to the raw materials, additives added and manufacturing processes. Water contact angle measurements have been widely used to evaluate surface wettability, and the surface hydrophobicity is a key parameter for characterising the surface interaction with virus particles^32–34^. We therefore investigated the hydrophobicity of the test material surfaces via comparing their water contact angles under ambient condition. As we expected, the hydrophobicity of material surfaces was determined mainly by their primary polymer components. However, we did not find any direct correlation between the materials water contact angle and their adsorption strength against pseudo-viruses. This is anticipated as viral attachment to manmade surfaces is a function of a series of physicochemical properties of the material^10,22,35^.

In addition, there are studies suggesting that surface chemistry may control virion adsorption strength and hence result in the reduced viable lifetime of virus attached to surfaces^32,33,35^. We therefore decided to determine the surface chemistry for all the test materials using ToF–SIMS. It is worth noting that other than the primary polymer components, a series of additives are used in the manufacturing process, the type and amount of which can vary. The most commonly used additives in polymer product manufacturing are plasticizers, surfactants, antioxidants, antistatic agents, pigments, flame retardants and catalysts^36^. Typically, dispersing agents are used in glove manufacturing to prevent the solid particles from gathering in latex dispersion^37^. In addition, thiurams, mercaptos and carbamates are found to be the most allergenic groups in additives of both natural and synthetic gloves^27^. However, access to the detailed material information for PPE products from the manufacturers was not possible due to fear of market competition^27^. In all cases, our ToF–SIMS analysis highlights that the PPE surfaces are not exclusively the named material but rather a composite of that, additives and surfactants left over from the items’ fabrication process.

**Figure 5:**
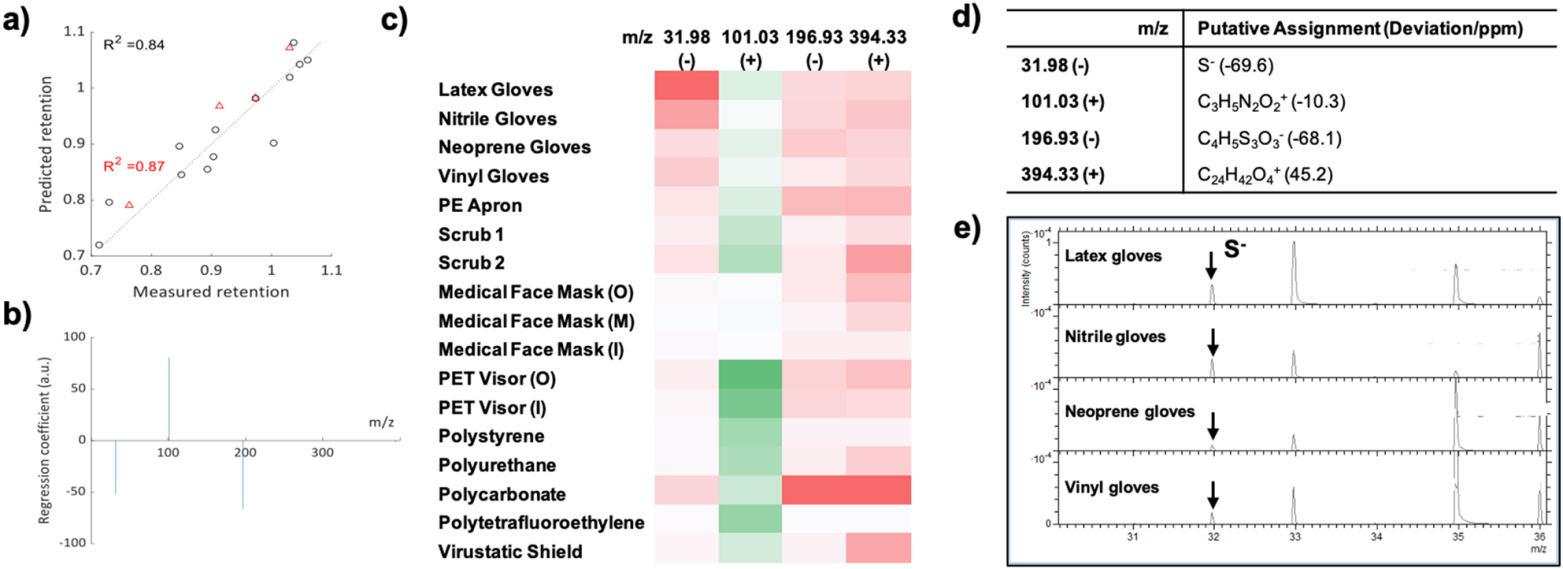
Correlation of the surface chemistries represented in the ToF–SIMS. PLS regression model used to predict the viral retention of materials by correlating pseudo-virus particle fluorescence intensity with the ToF–SIMS spectra. (**a**) The predicted viral retention determined from regression model (R^2^ = 0.87) compared to the measured retention (R^2^ = 0.84) The y = x line is drawn as a guide. (**b**) The key ions identified to be important by ToF–SIMS PLS regression analysis for viral retention. (**c**) The intensity of the key ions of the test materials shaded according to the value (dark to white: high to low intensity; the ion that promotes viral retention is in green, while the ions that resist to viral retention are in red) (**d**) Putative assignments of the key ions (**e**) S^−^ peaks in ToF–SIMS negative spectra of the four types of gloves.

ToF–SIMS analysis combined with Partial Least Square (PLS) regression has been demonstrated as a powerful tool for corelating surface chemistry represented in mass spectra with a univariate data set such as bacterial attachment to surface^38,39^. This method was used here to unveil correlations between the surface chemistry of the test materials and viral retention represented by mean fluorescence intensity, and to identify key surface moieties for viral attachment. The data collected from all the 17 materials were split into training and test sets (13:4), and LASSO regression was used to remove uninformative descriptors, and PLS performed with 2 latent variables (minimise root mean square error of cross-validation). The PLS regression model produced by this analysis successfully predicted viral retention from the ToF–SIMS spectra, as evidenced by the linear relationship between predicted and measured retention with an R^2^ value of 0.84 and 0.87 for the training and test sets (Fig. 5a). This indicates that the surface chemistry of the PPE as measured by ToF–SIMS partially correlates with the strength of viral adsorption to a material. Four key ions, which gives the highest influence on viral retention quantified by the absolute value of PLS regression coefficients, were identified, where three negative values at m/z 31.98 (−), 196.93 (−) and 394.33 (+) mean these ions correlated with resistance to viral retention, while the positive value at m/z 101.03 (+) means this ion correlated with promotion of viral retention (Fig. 5b). The relative intensity of the four key ions in ToF–SIMS spectra for the test materials are shown in Fig. 5c, where darker colour indicates higher signal intensity. Putative assignments of these four key ions are listed in Fig. 5d, which were selected by their low deviation from the peak position and their chemical plausibility. (Table S2) The negative ion at m/z 31.98 was assigned to S^−^, (Fig. 5e) which correlated to the low viral retention of the materials with sulfur containing ions identified in the ToF–SIMS analysis, including latex gloves, nitrile gloves, vinyl gloves and PC. The ion at m/z 196.93 (−) correlated to the low viral retention of PC, PE apron, neoprene gloves, nitrile gloves and latex gloves, the assignments of which aligned with the existence of sulfur containing components that were possibly from the addition of common manufacturer additives. The ion at m/z 394.33 (+) correlated to the low viral retention of PC, Scrub 2, Virustatic shield and PE apron, and possibly from the addition of surfactants with a long hydrocarbon chain and hydrophilic moiety. The ion peak at m/z 101.03 (+) were found in PET visor (O), PET visor (I), PS, PU and PTFE giving relatively high intensity, putatively assigned to a nitrogen containing ion, and correlated to the high viral retention. Again, the putative assignments of these ions indicate that common additives may be added in the manufacturer process. In general, PLS regression successfully demonstrated the correlation between four key ions: m/z 31.98 (−), 101.03 (+), 196.93 (−) and 394.33 (+) identified in ToF–SIMS spectra and viral retention under air flow challenge. However, the produced model is limited for making future predictions due to (1) the low range of performance of the test materials, (2) the diversity of unknown components due to the manufacturer process and (3) the relatively limited type and number of materials used to construct the model.

For further fundamental investigation of intermolecular interactions of SARS-CoV-2 virions with materials, data collection from a large library of materials with a broad range of well-defined surfaces is desirable. High-throughput polymer microarray has been applied to establish such platform for the discovery of novel materials to control bacterial, fungal and stem cell surface colonisation and help to build associated theoretical frameworks^38,40–42^. The potential of high-throughput polymer microarray screening to identify materials for differential virus binding was descried in our previous study^22^, the methodology will be applied to the discovery and development of novel anti-SARS-CoV-2 to address the understanding of the interactions between the virions and polymer surfaces..

## Conclusions

The antiviral efficacy of current commercially available PPE and commonly used polymer surfaces have been evaluated with live SARS-CoV-2 at 10 min and 30 min contact time under ambient condition, chosen to represent realistic residence times in general usage. An antiviral material was defined as a material that resulted in at least a tenfold reduction in the SARS-CoV-2 titre in a cell based viability assay. Other than an antiviral protein-coated shield fabric, none of the existing simple polymer-based PPE materials exhibited significant antiviral activity against SARS-CoV-2 for these contact times.

The possibility of spread of the viruses from contaminated material surfaces was assessed using a pseudo-virus model of SARS-CoV-2 with a laminar air flow system used to mimic the scenario of air flow generated by movement and doffing in a real-world setting. The results indicate that the pseudo-virus can be removed/resuspended more easily from polycarbonate, latex gloves and nitrile gloves than from other test materials.

To better understand the difference of viral attachment and inactivation at material surfaces in terms of material physicochemical properties, water contact angles of the test materials were measured and the surface chemistry of the test materials were examined using ToF–SIMS. No direct correlation was found between the material surface wettability and viral retention under air flow challenge. The surface chemistry of these commercial polymer surfaces was determined to have large contribution from additives and surfactants related to their fabrication and packaging processes. PLS regression modelling successfully demonstrated a correlation between the surface chemistry and viral retention.

## Supplementary Information

Supplementary Information.

## Data Availability

The datasets generated and/or analysed during the current study are available in the Nottingham Research Data Management Repository at https://www.nottingham.ac.uk/dts/researcher/managing-data/research-data-repository.aspx (https://doi.org/10.17639/nott.7205).
