# Supplementary material for: Evaluation of the relative potential for contact and doffing transmission of SARS-CoV-2 by a range of personal protective equipment materials
Source: Sci Rep. 2022 Oct 5;12:16654. doi: 10.1038/s41598-022-20952-8 (PMC9533983; doi:10.1038/s41598-022-20952-8)
Supplement: Supplementary file 1 — Supplementary Information. [file 41598_2022_20952_MOESM1_ESM.docx]

**Evaluation of the relative potential for contact and doffing transmission of SARS-CoV-2 by a range of personal protective equipment materials**

- **Supplementary Information**

Xuan Xue^1^, Christopher M. Coleman^2, 3^, Joshua D. Duncan^2^, Andrew L. Hook^1^, Jonathan K. Ball^2, 3, 4^, Cameron Alexander^5^ and Morgan R. Alexander^1^*

^1^ Division of Advanced Materials and Healthcare Technologies, School of Pharmacy, University of Nottingham, NG7 2RD, UK.

^2^ School of Life Sciences, ^3^ Wolfson Centre for Research on Global Virus Infections, ^4^ Nottingham Biomedical Research Centre, University of Nottingham, Queen’s Medical Centre, NG7 2UH, UK

^5^ Division of Molecular Therapeutics and Formulation, School of Pharmacy, University of Nottingham, Nottingham, NG7 2RD, UK.

*Corresponding Author: morgan.alexander@nottingham.ac.uk (M.R.A)

| **Material** | **Water contact angle (°)** | **Material** | **Water contact angle (°)** | **Material** | **Water contact angle (°)** |
| --- | --- | --- | --- | --- | --- |
| Latex | 86.3 ± 4.7 | Face mask (O) | 85.6 ± 7.8 | PS | 88.7 ± 2.5 |
| Nitrile | 87.7 ± 2.3 | Face mask (M) | 77.9 ± 4.6 | PU | 86.7 ± 1.5 |
| Neoprene | 85.7 ± 2.9 | Face Mask (I) | 76.6 ± 8.8 | PC | 55.5 ± 1.0 |
| Vinyl | 71.7 ± 1.5 | PET visor (O) | 21.3 ± 3.2 | PTFE | 89.7 ± 5.0 |
| PE apron | 87.0 ± 1.0 | PET visor (I) | 16.7 ± 1.5 |  |  |
| Scrub 1 | n/a | Virustatic Shield | 93.2 ± 2.9 |  |  |
| Scrub 2 | n/a |  |  |  |  |

**Table S1.** Water contact angles of the test materials measured under ambient condition at room temperature.

| **m/z** | **Putative Assignment (Deviation/ppm)** |
| --- | --- |
| **31.98 (-)** | S^-^ (-69.6) |
| **101.0332 (+)** | C_3_H_5_N_2_O_2_^+^ (-10.3), C_4_H_7_SN^+^ (41.0), C_8_H_5_^+^ (-50.1), C_7_H_3_N^+^ (74.4), C_5_H_9_S^+^ (-83.4), C_4_H_5_O_3_^+^ (100.9), C_5_H_9_O_2_ (-259.1) |
| **196.9271 (-)** | H_5_S_3_O_6_^-^ (9.4), C_4_H_5_S_4_O^-^ (22.1), C_3_HS_2_O_6_^-^ (26.5), C_4_H_5_S_3_O_3_^-^ (-68.1), H_5_S_2_O_8_^-^ (-80.8), CH_9_S_4_O_3_^-^ (-85.2), H_5_S_4_O_4_^-^ (99.6) |
| **394.3256 (+)** | C_24_H_42_O_4_^+^ (45.2), C_25_H_46_O_3_^+^ (-47.1), C_22_H_43_O_4_Na^+^ (51.3) |

**Table S2.** Putative assignments of the four key ions and their deviations to indicate the selection.


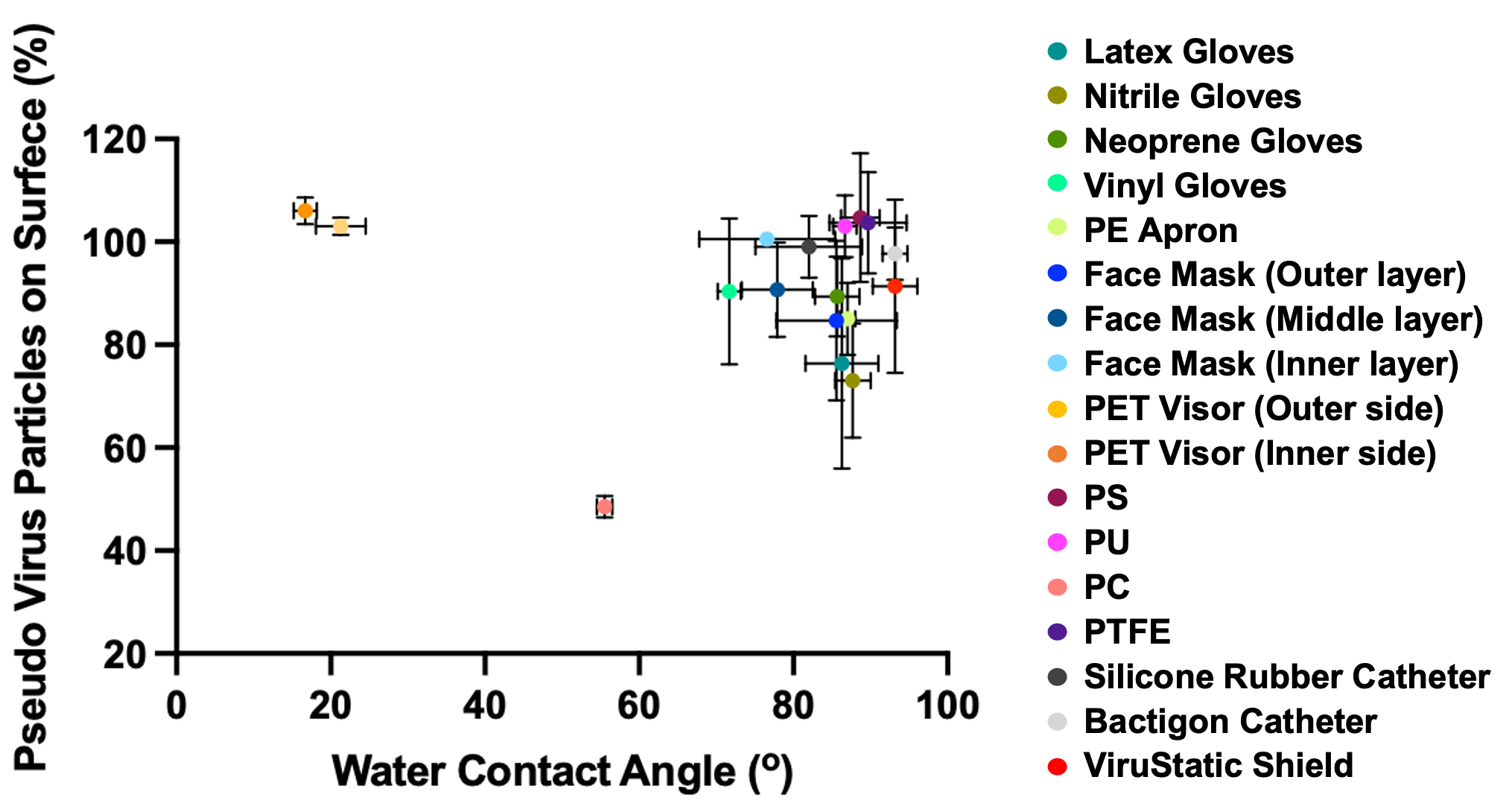


**Figure S1.** The scatter plot of the correlation between adsorption strength of the materials against pseudo virus particles and material water contact angle.


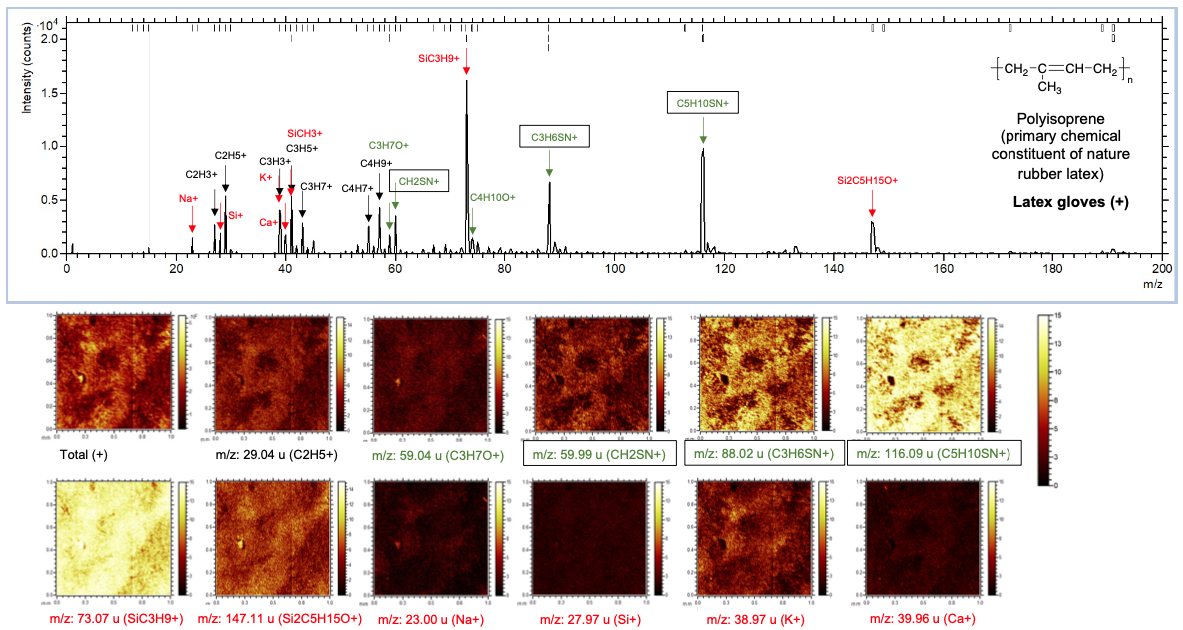


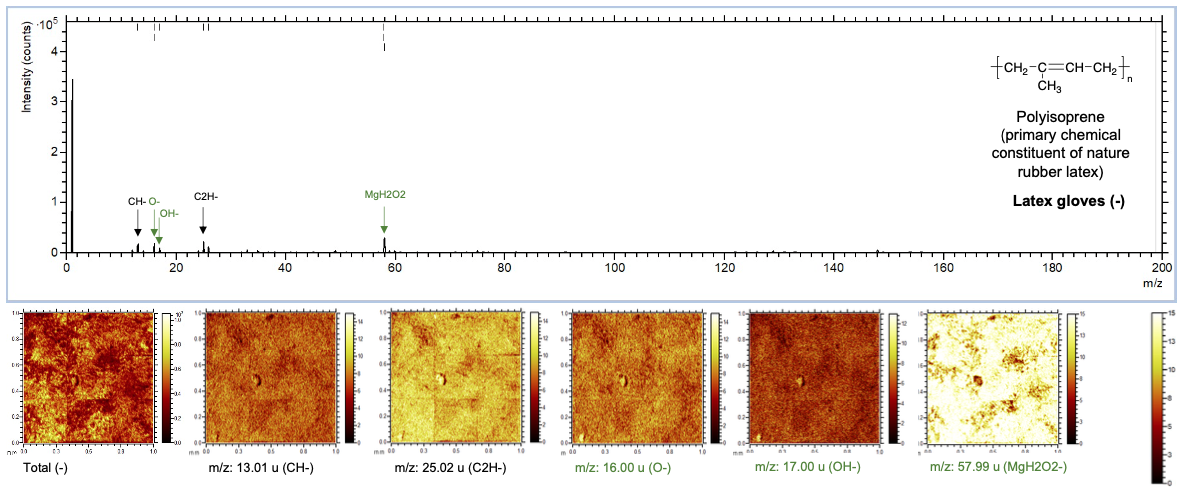


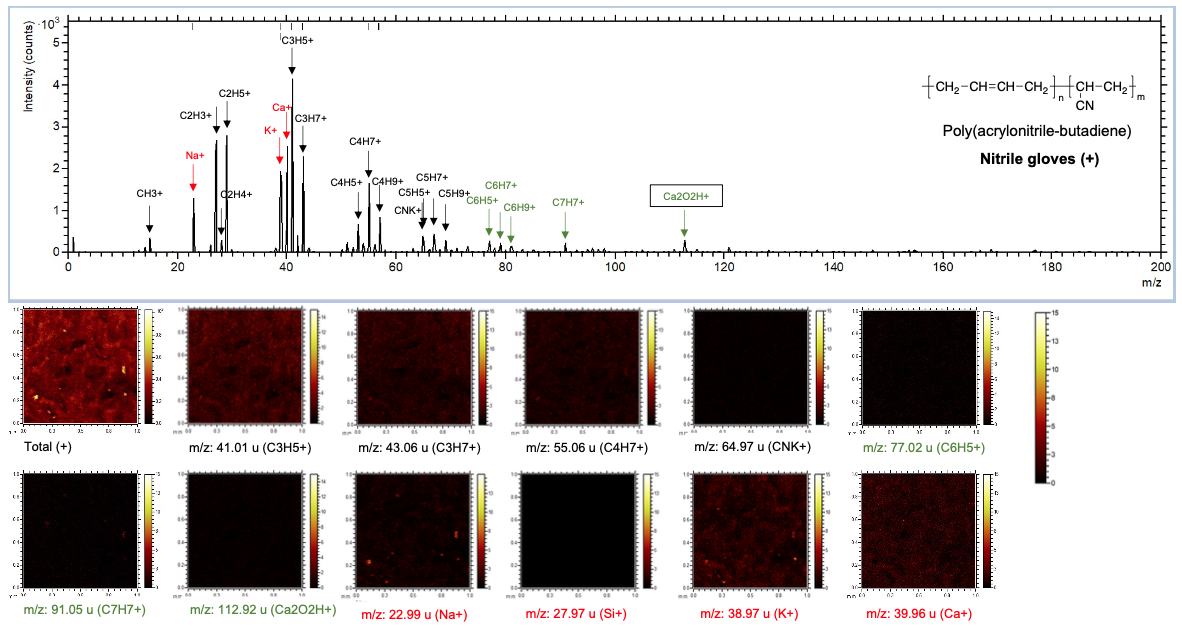


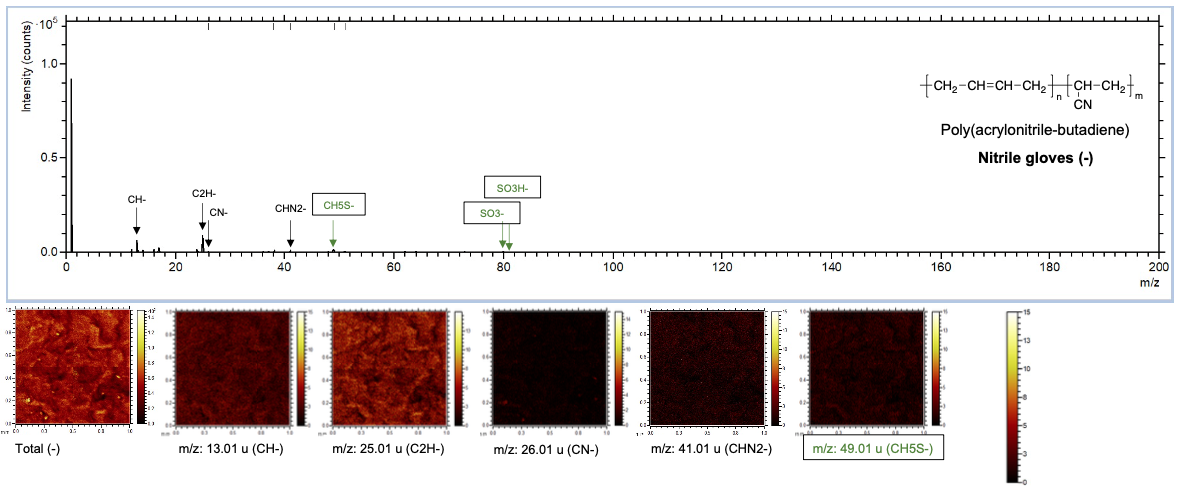


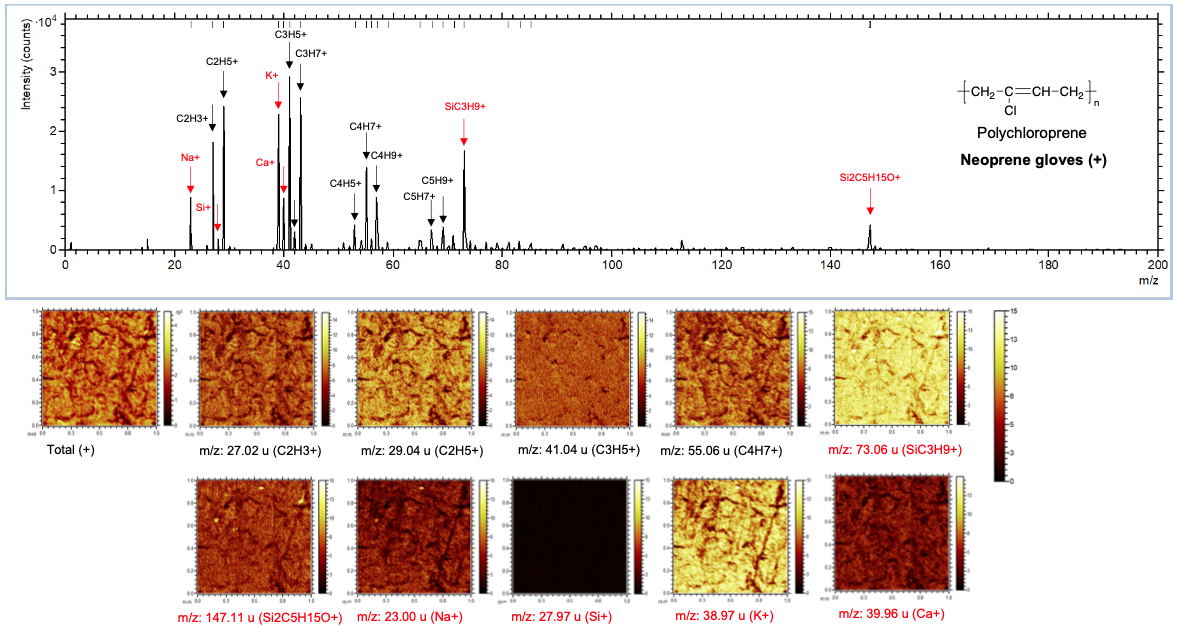


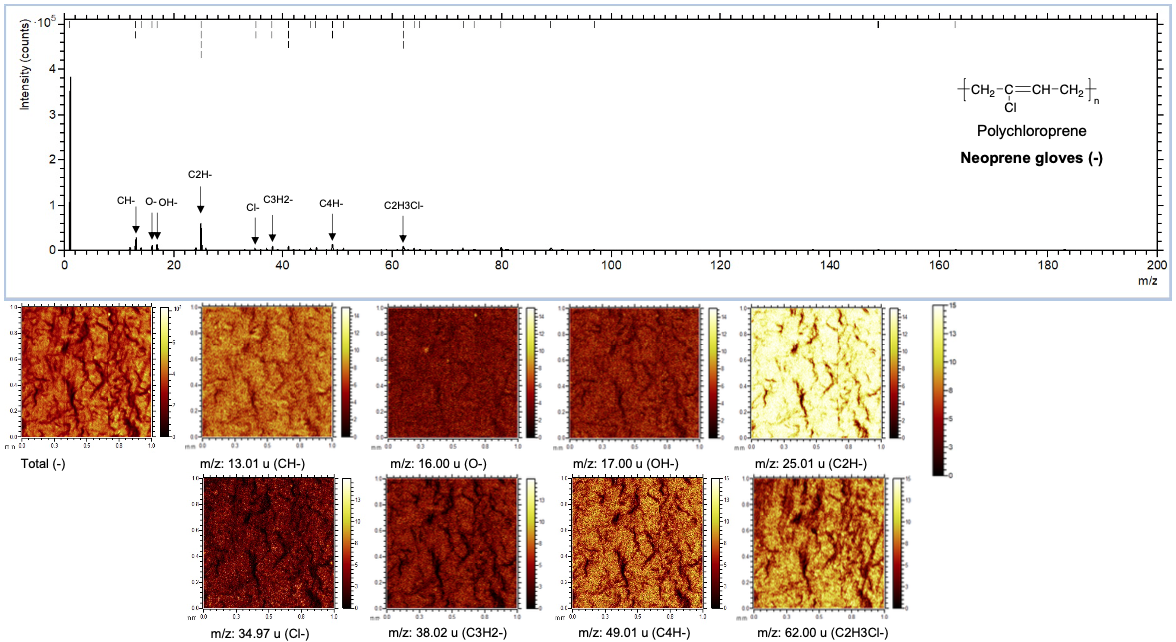


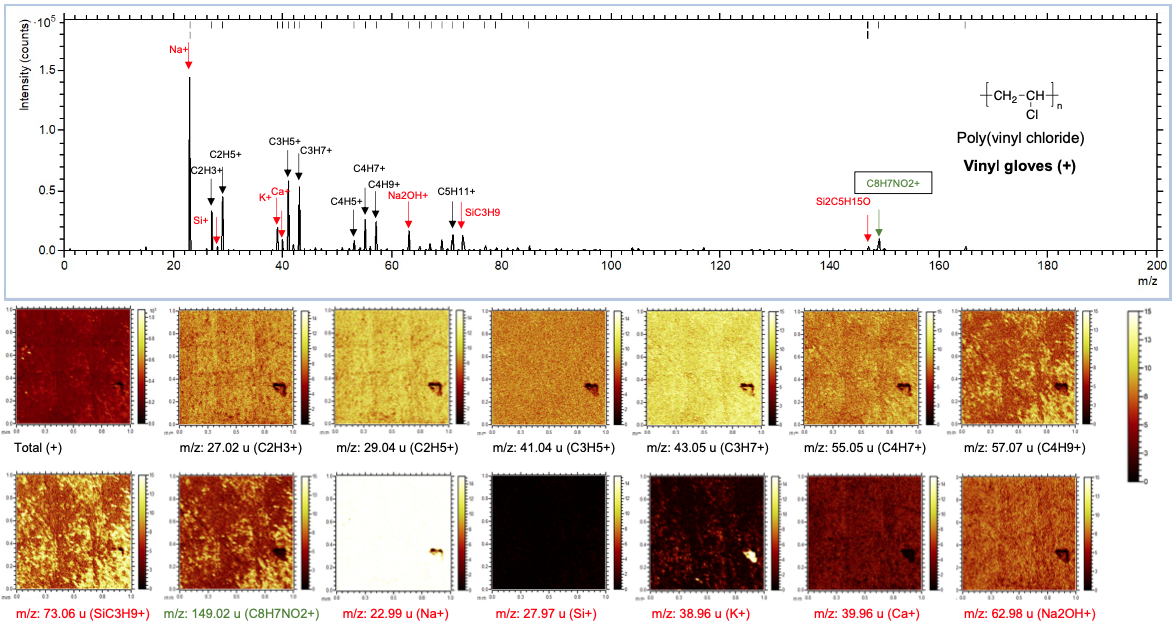


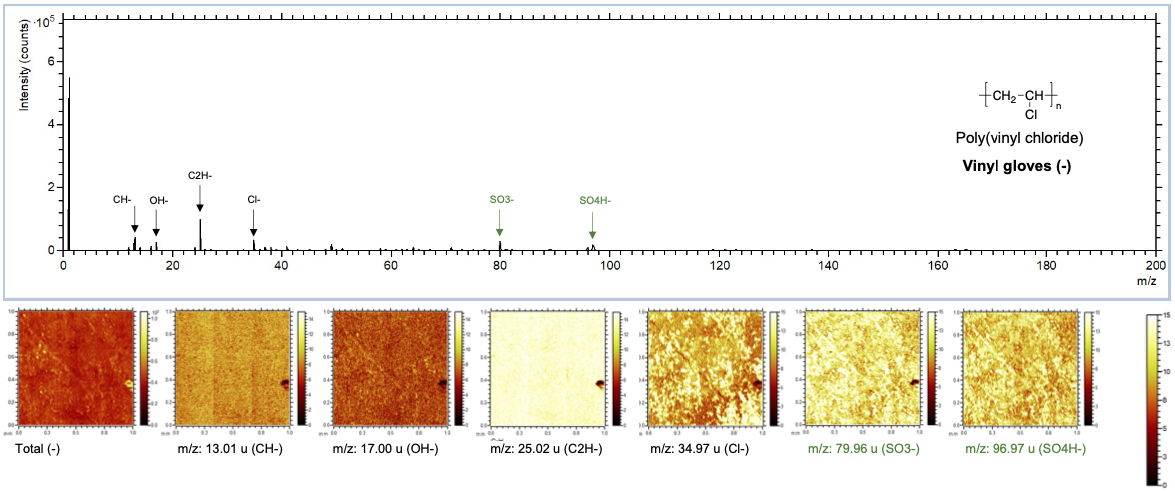


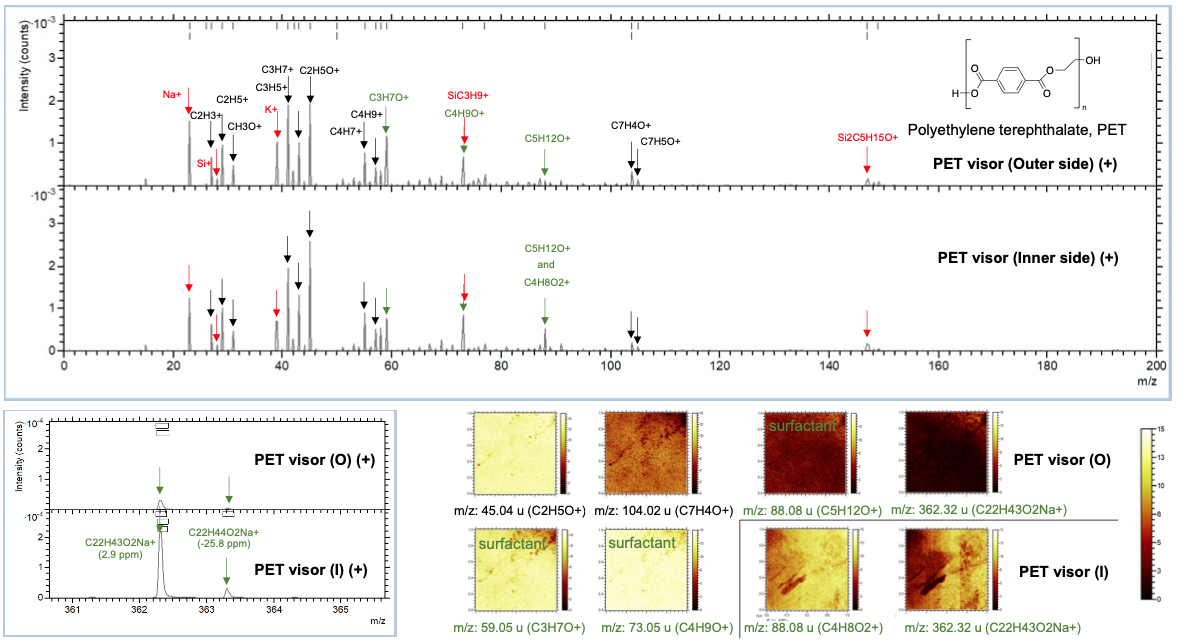


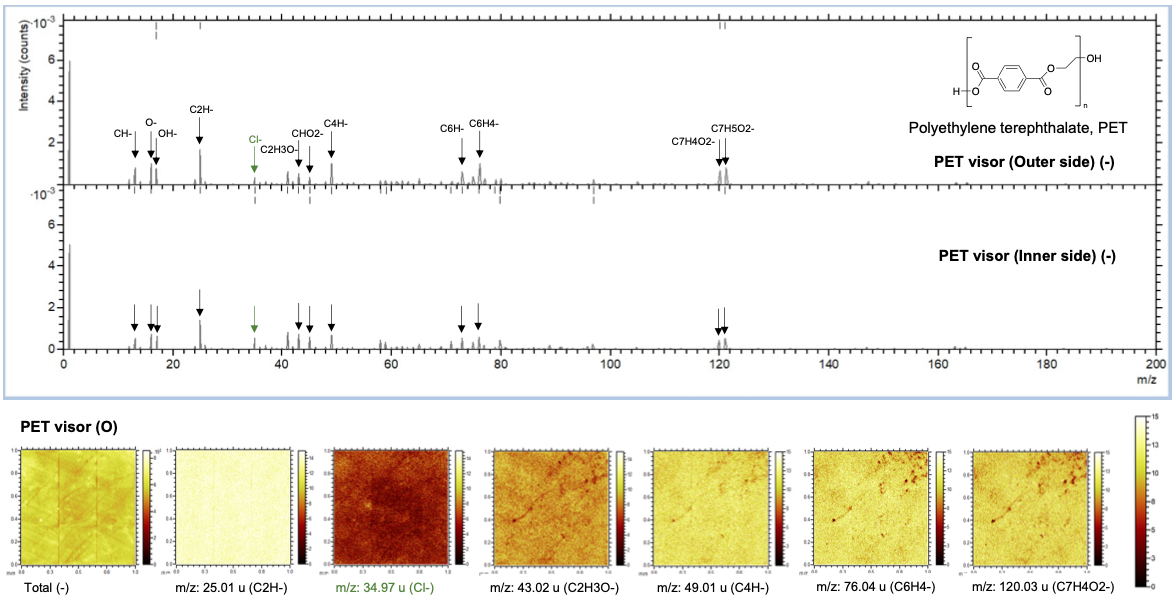


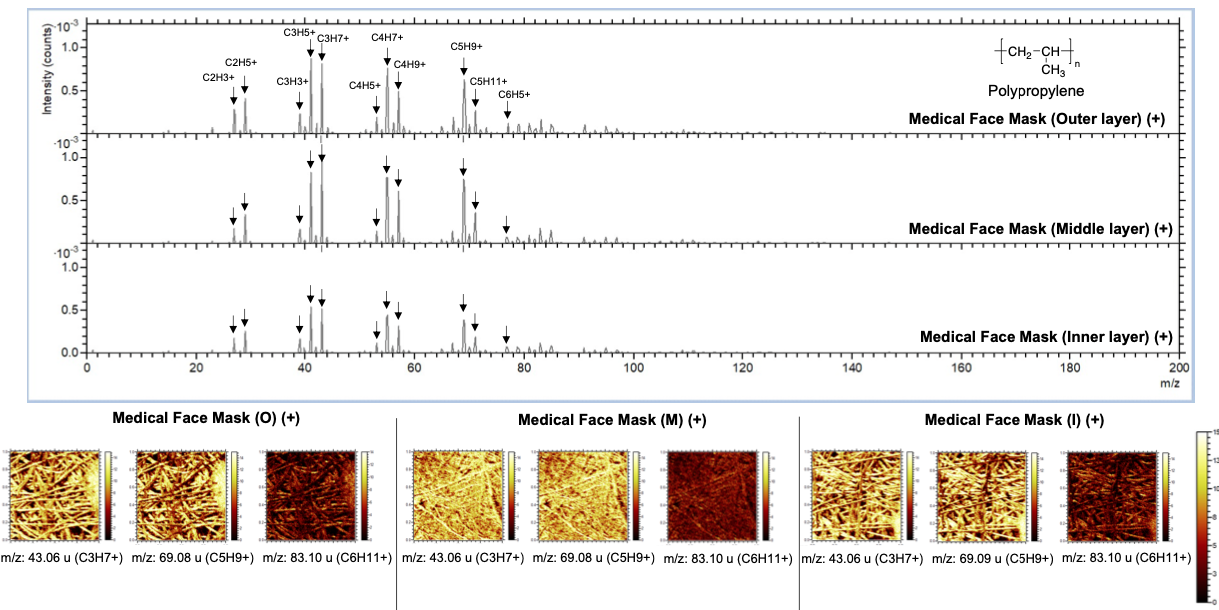


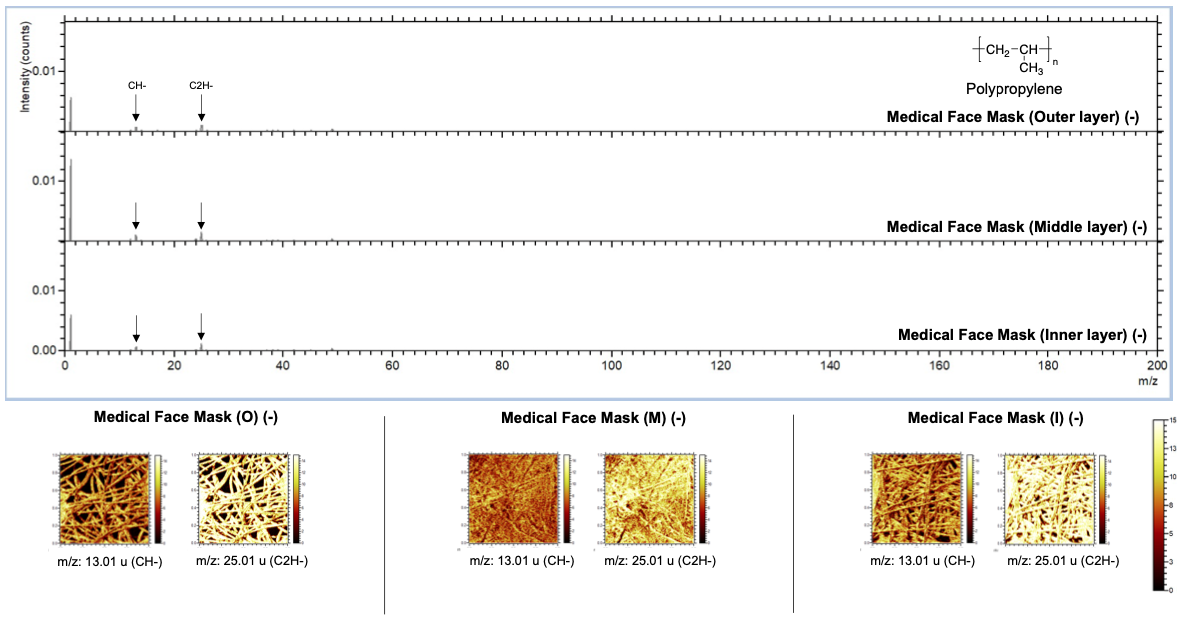


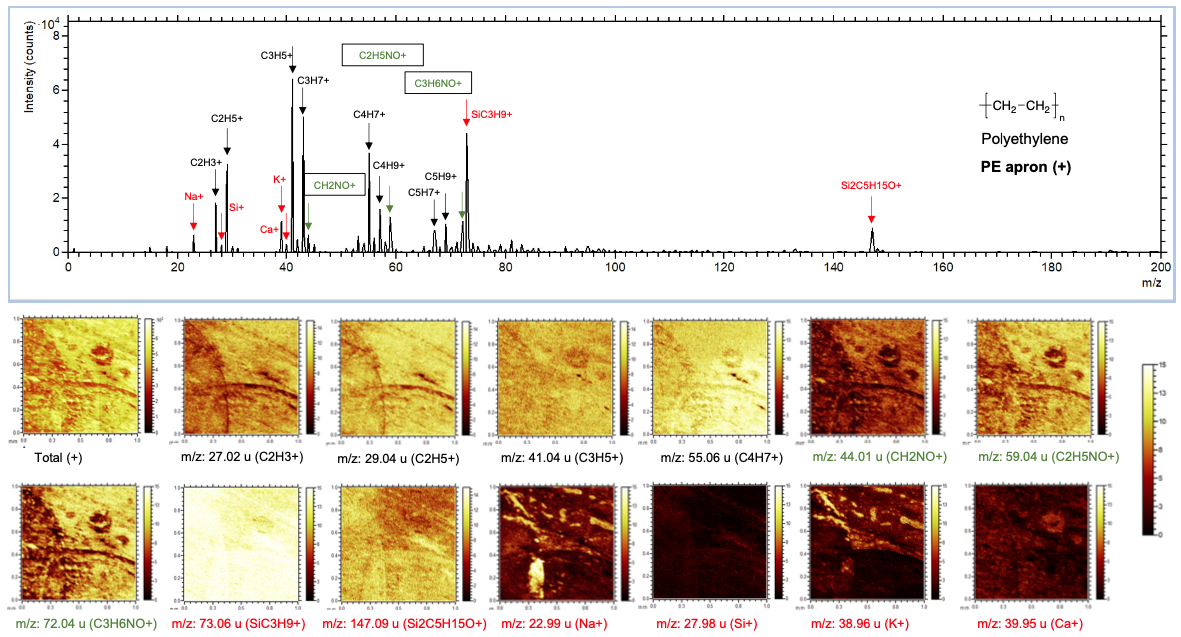


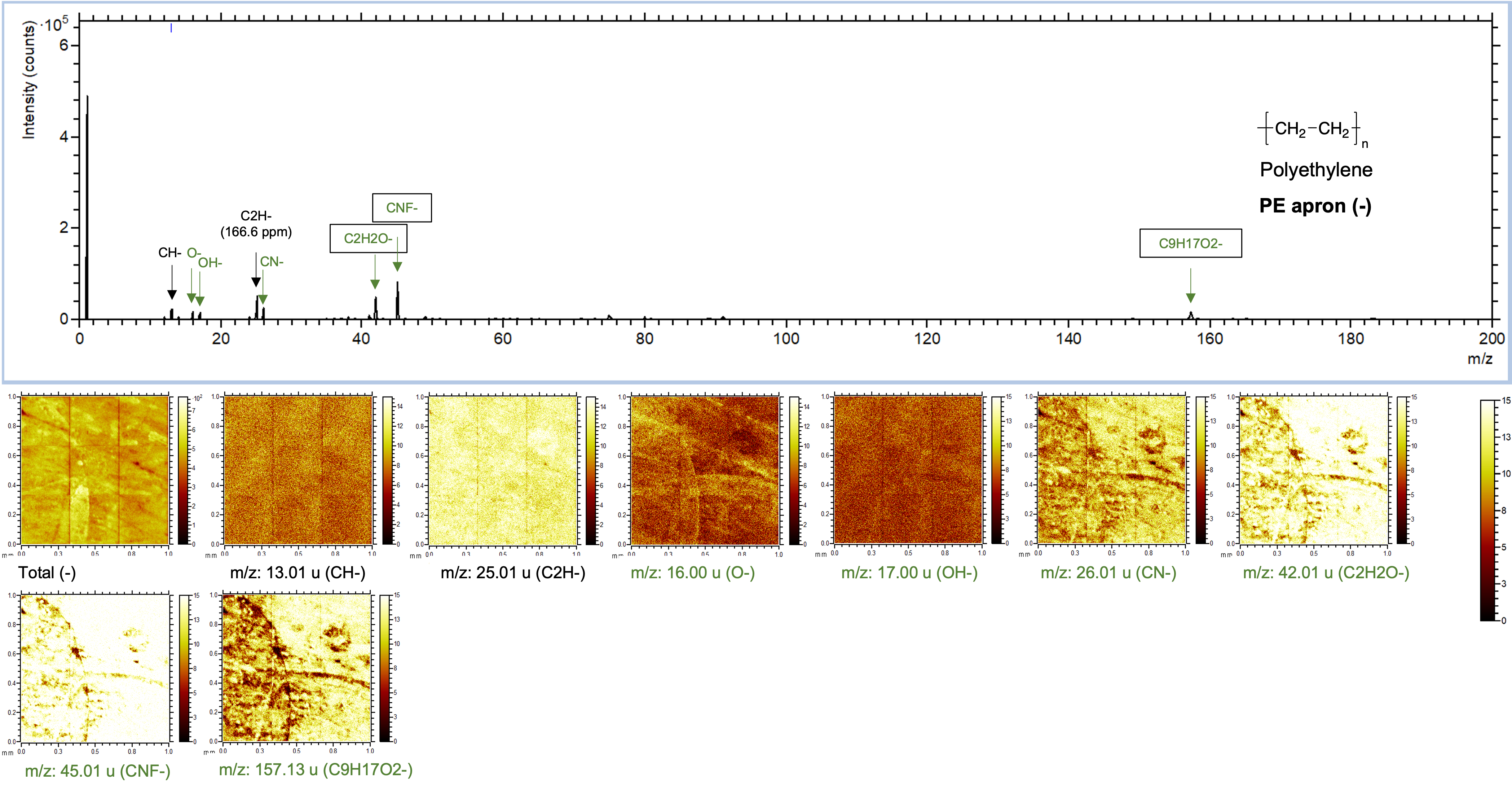


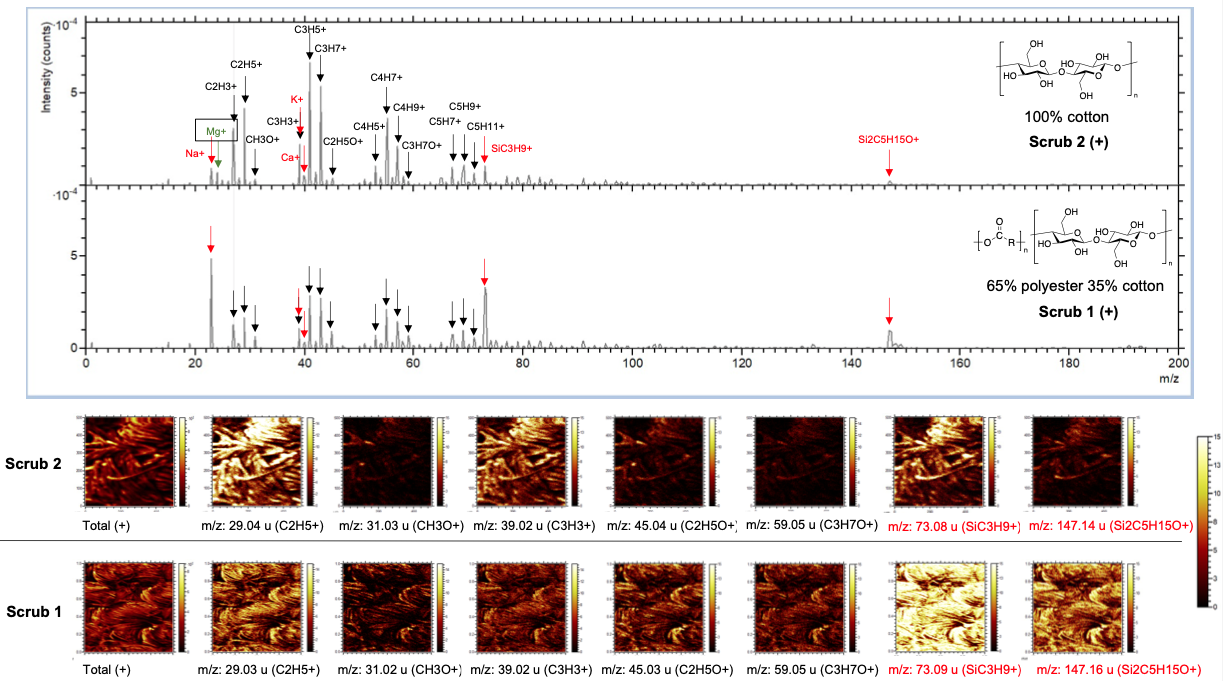


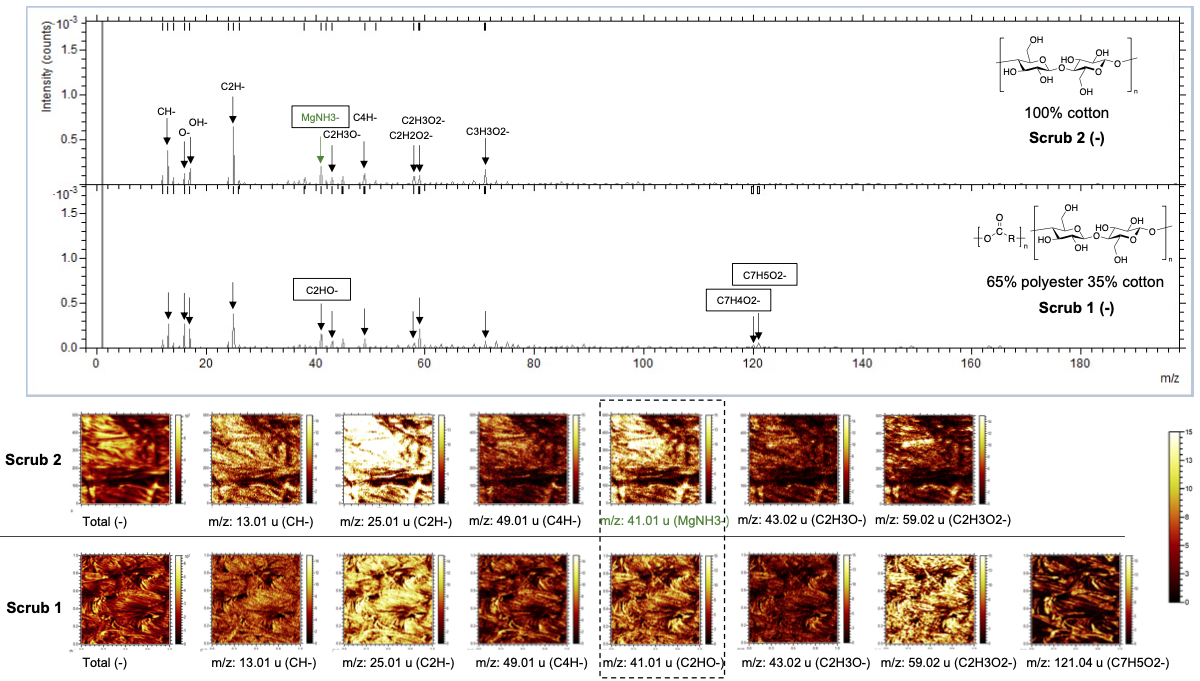


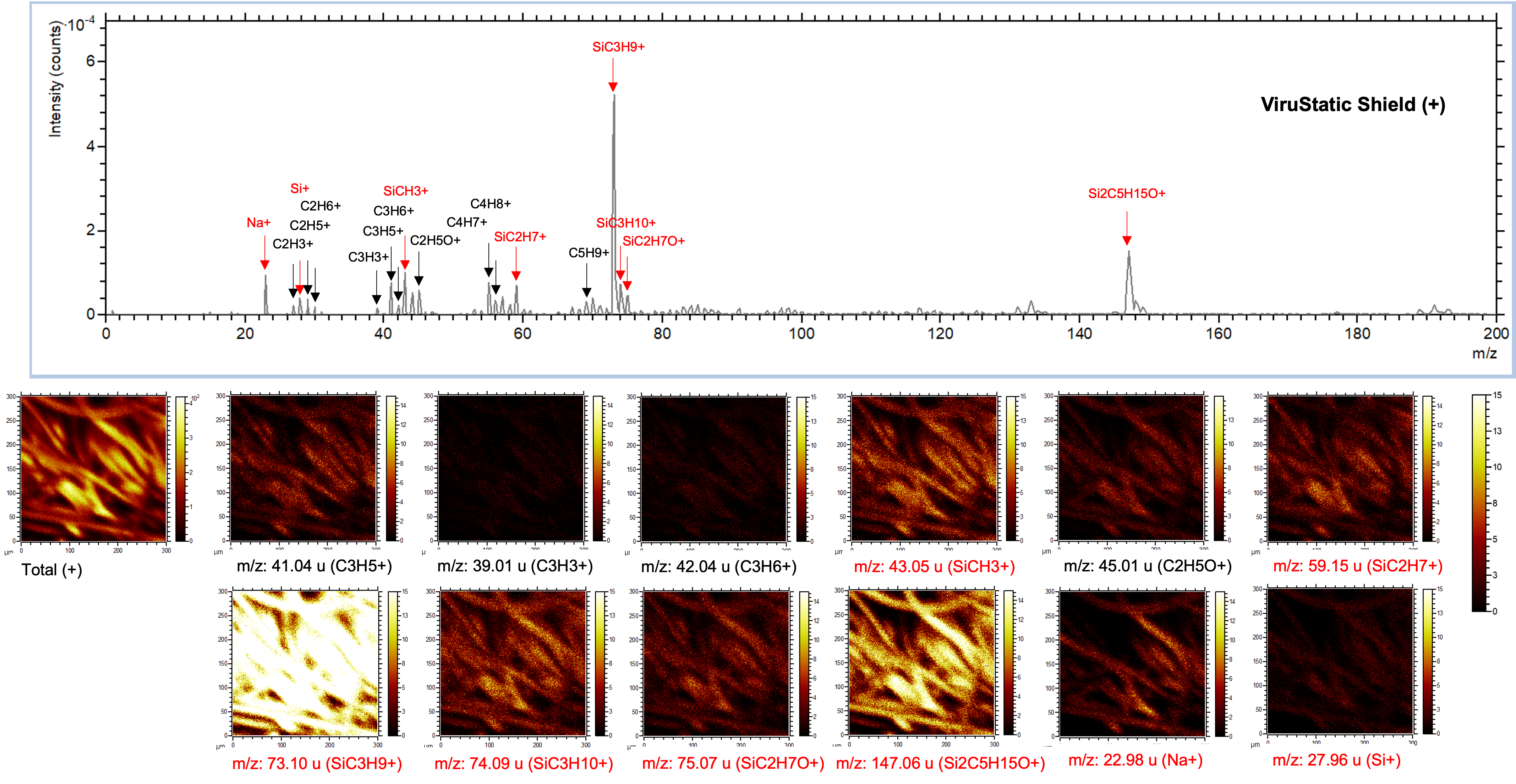


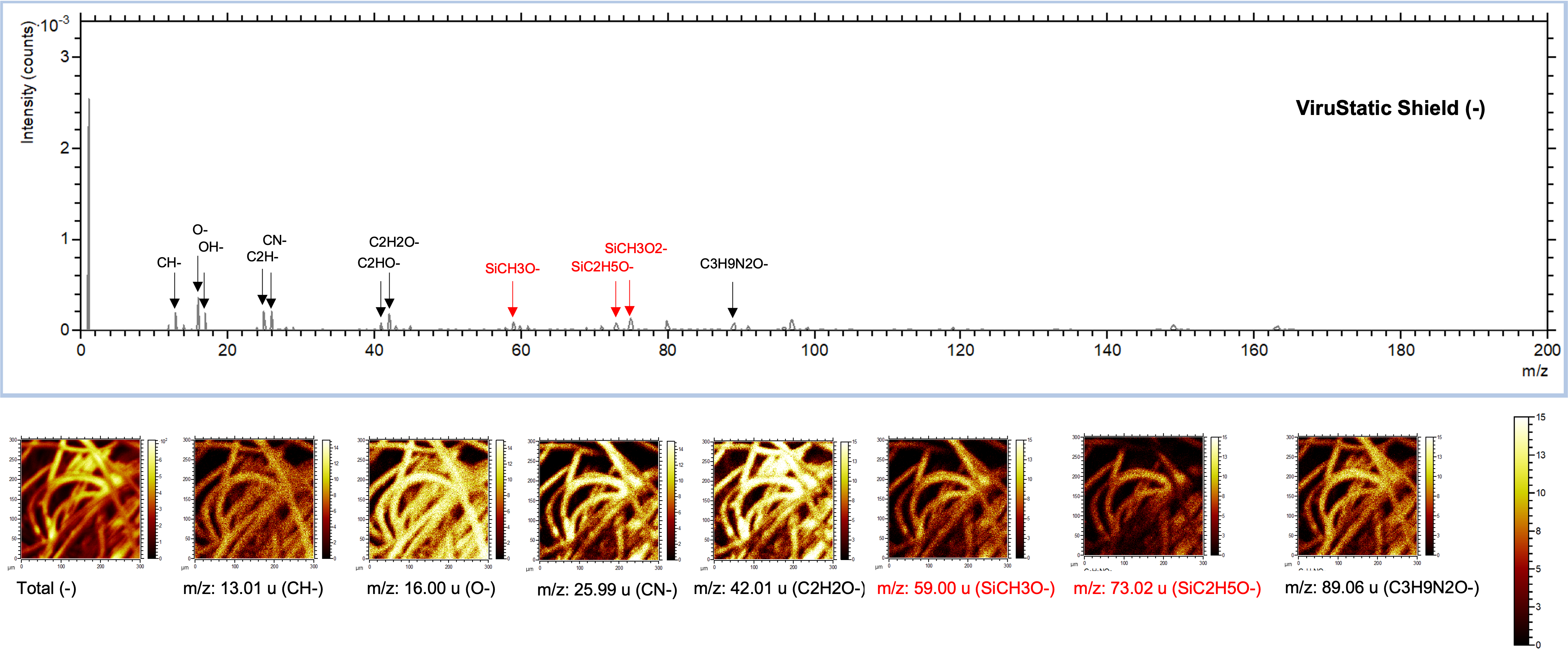


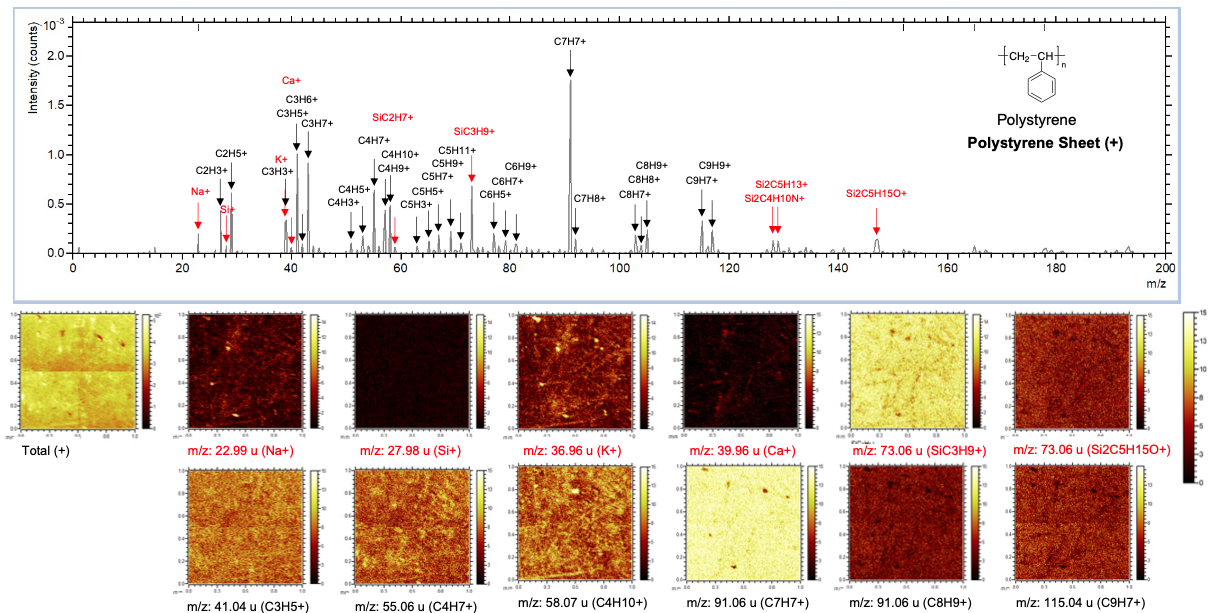


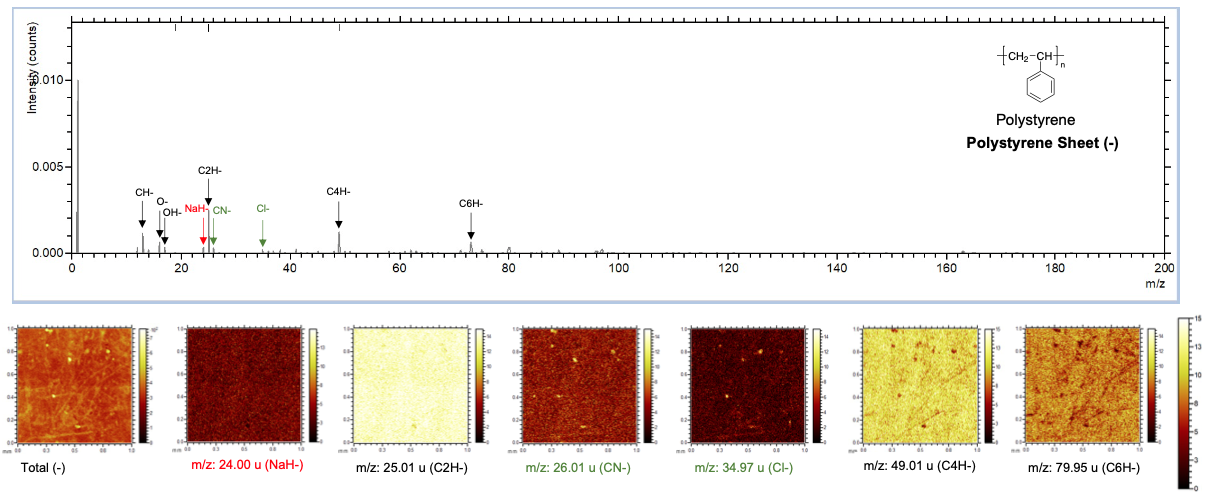


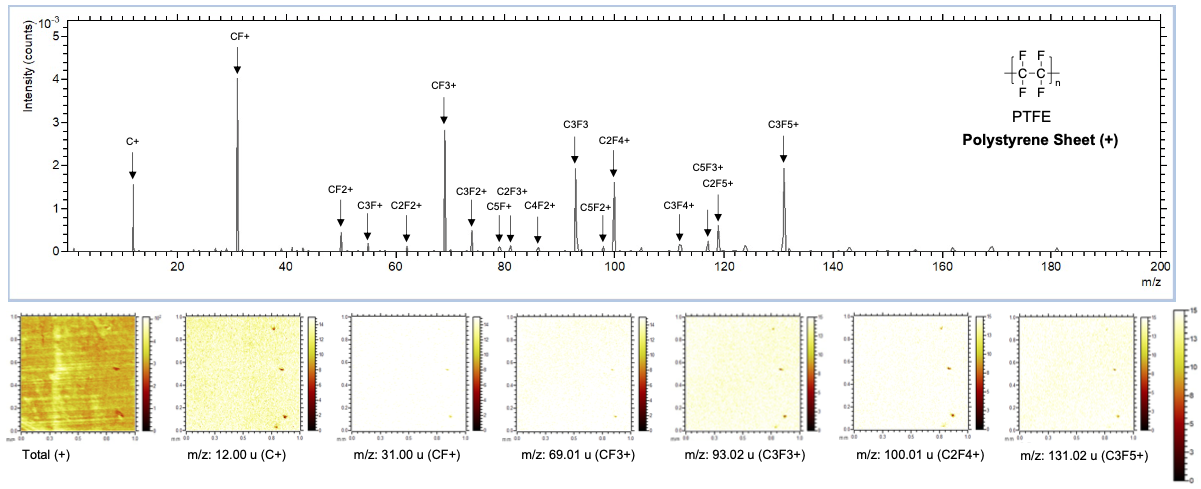


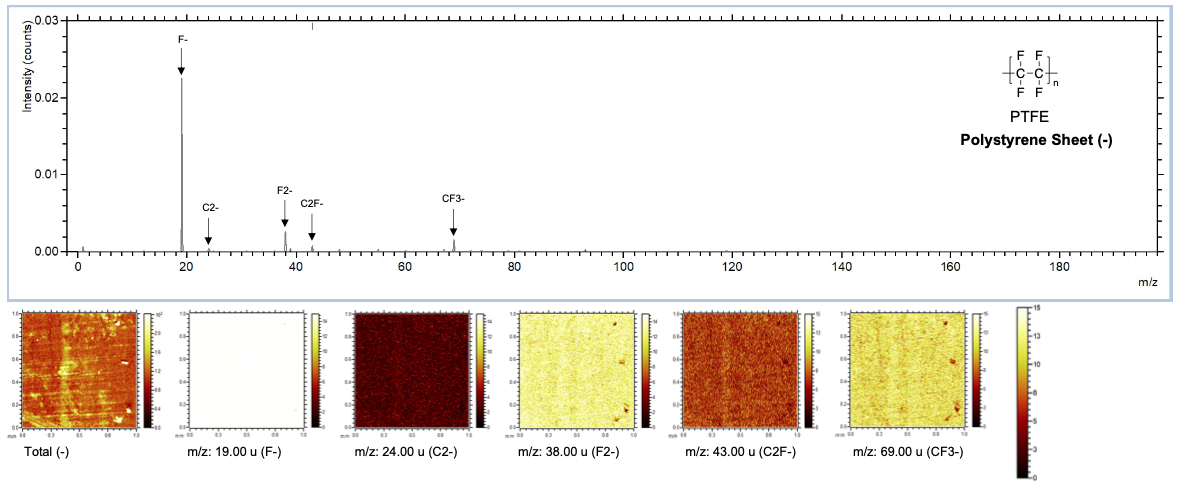


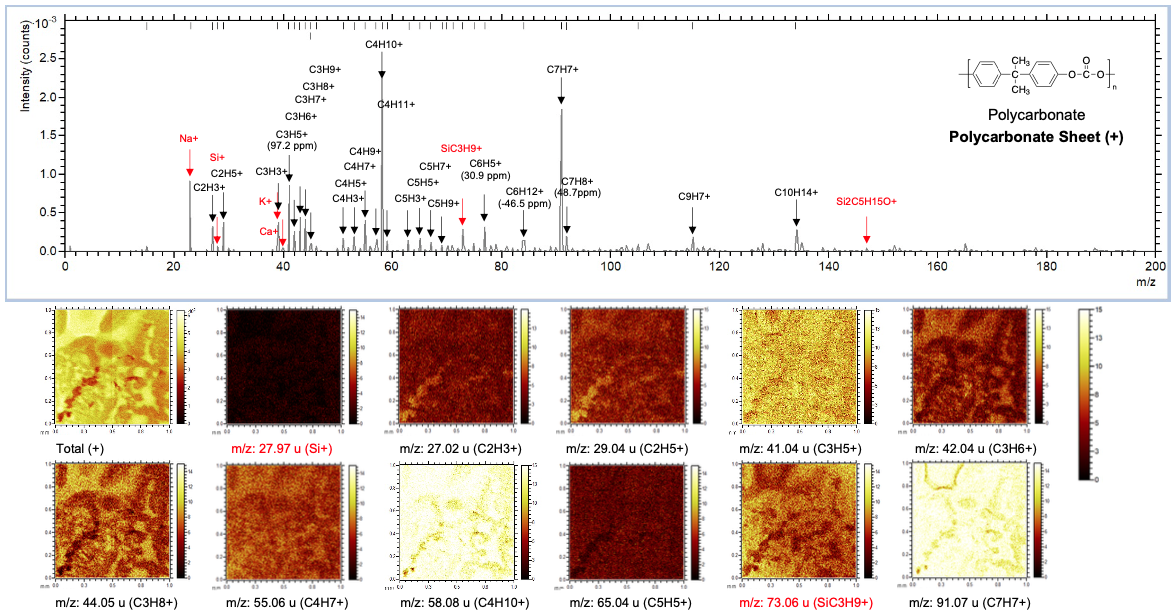


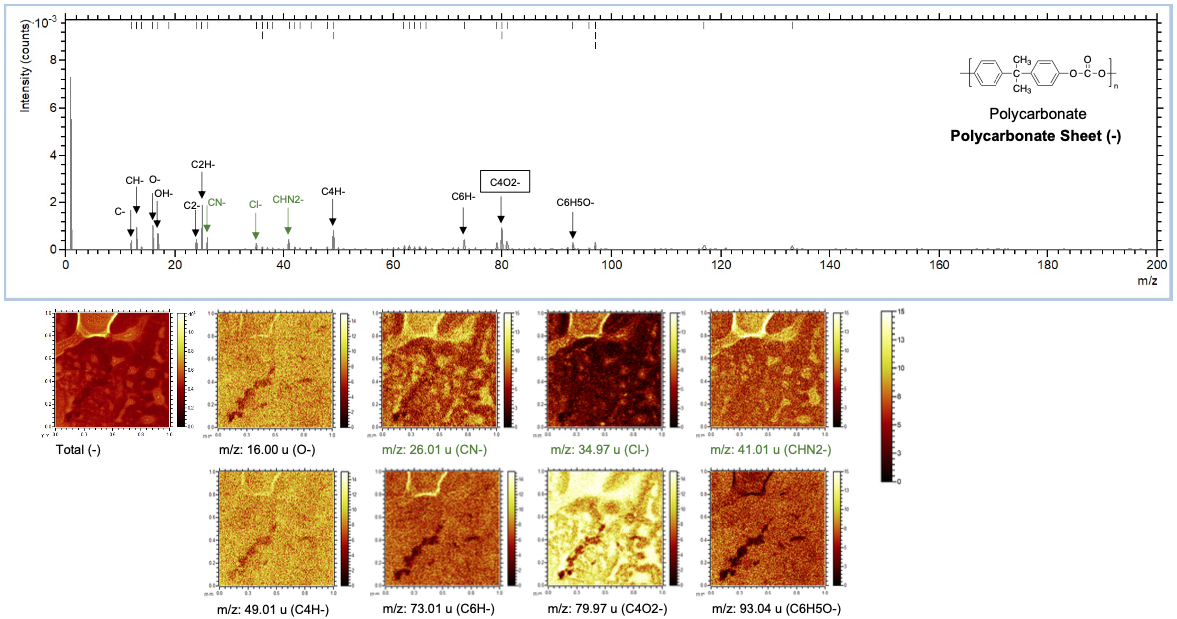


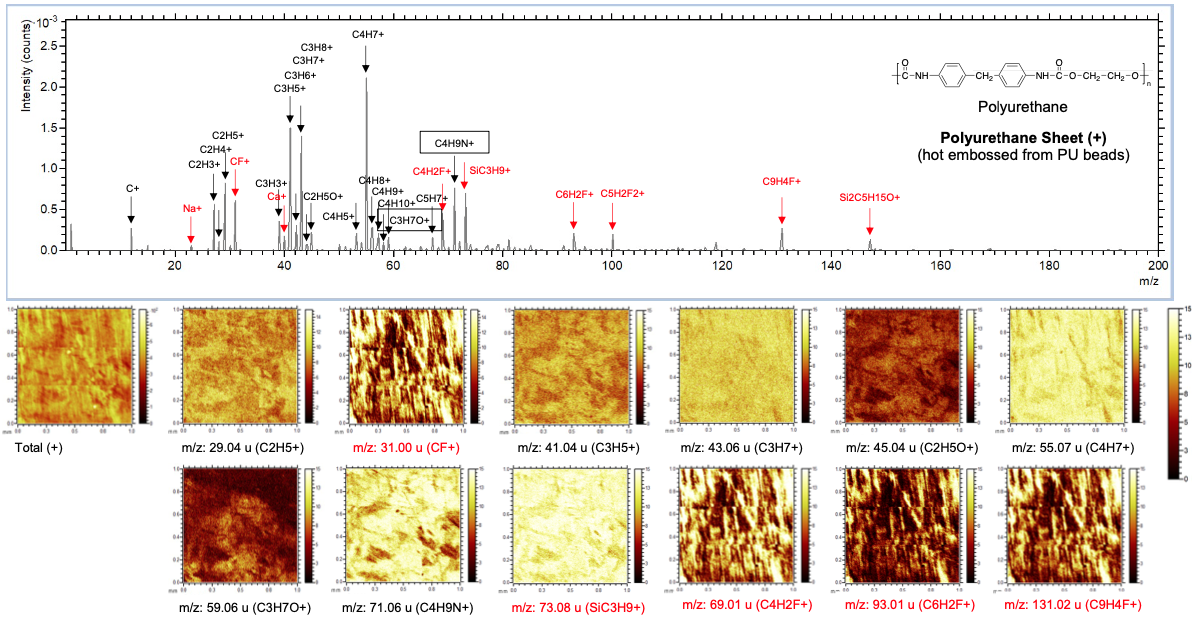


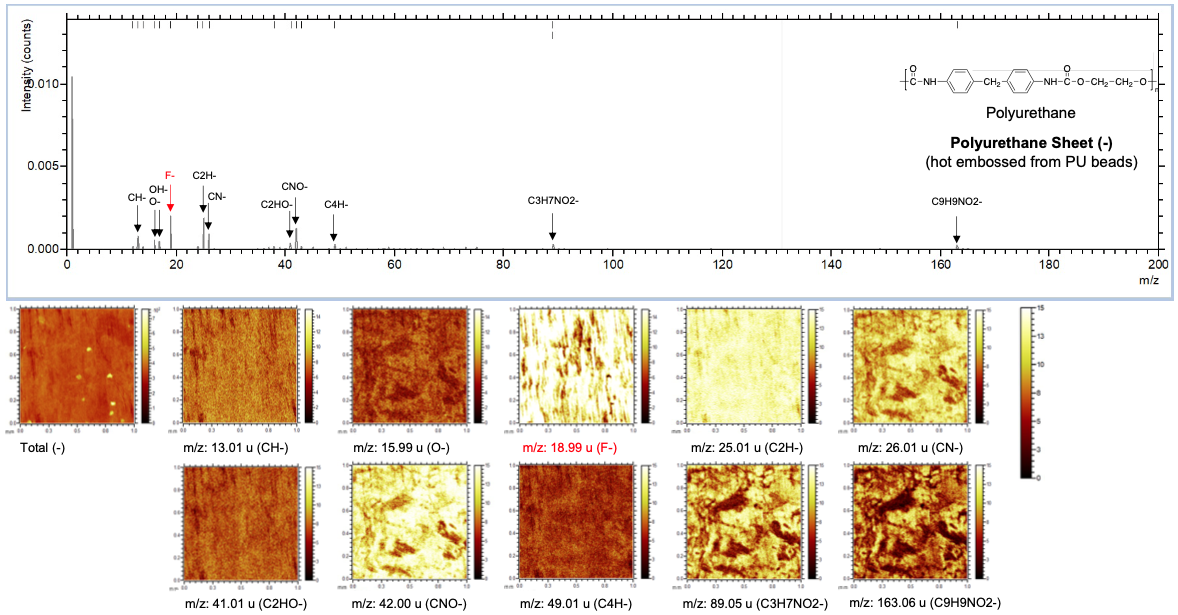


**Figure S2**. ToF-SIMS spectra and ion images for all the test materials (x17)
